# Alpha-Tocopherol, a Powerful Molecule, Leads to the Formation of Oxylipins in Polyunsaturated Oils Differently to the Temperature Increase: A Detailed Study by Proton Nuclear Magnetic Resonance of Walnut Oil Oxidation

**DOI:** 10.3390/antiox11040604

**Published:** 2022-03-22

**Authors:** Sofía del Caño-Ochoa, Ainhoa Ruiz-Aracama, María D. Guillén

**Affiliations:** Food Technology, Faculty of Pharmacy, Lascaray Research Center, University of the Basque Country (UPV-EHU), Paseo de la Universidad n 7, 01006 Vitoria-Gasteiz, Spain; sofia.delcano@ehu.eus (S.d.C.-O.); ainhoa.ruiz@ehu.eus (A.R.-A.)

**Keywords:** polyunsaturated edible oils, walnut oil, alpha-tocopherol, accelerated storage, ^1^H NMR, oxidative stability, oxidation process, oxylipins

## Abstract

Lipid oxidation causes food degradation and the formation of toxic compounds. Therefore, the addition to foods of compounds able to avoid, delay or minimize this degradative process is a commonly used strategy. Nevertheless, neither the identity of most of the formed compounds in this complex process nor the way in which their formation is affected by the strategy used are well known. In this context, the effect the temperature increase and the enrichment level in alpha-tocopherol on the evolution of the walnut oil oxidation, as a model of an oil rich in polyunsaturated omega-6 acyl groups, submitted to storage conditions, are tackled by ^1^H NMR. The study has allowed knowing the degradation kinetic of both the oil acyl groups and alpha-tocopherol, the identification of a very high number of oxylipins and the kinetic of their formation. The temperature increase accelerates the formation of all oxylipins, favouring the formation of hydroperoxy conjugated *E*,*E*-dienes and related derivatives versus that of the *Z*,*E*-isomers. The enrichment in alpha-tocopherol accelerates the formation of hydroperoxy conjugated *Z*,*E*-dienes and related derivatives, and delays in relation to the formation of the former that of the *E*,*E*-isomers and related derivatives, hindering, to a certain extent, the formation of the latter in line with the enrichment level.

## 1. Introduction

Lipid oxidation is one of the most important reactions that takes place in food. It is generally known that it produces the degradation of these macronutrients, generating toxic compounds and others with off-flavour, so this reaction has economic, nutritional and food safety repercussions. In addition, this reaction can also occurs endogenously during digestion and in cells and tissues, and the oxidation compounds formed are considered to be responsible for several diseases [1,2,3,4,5,6]. Nevertheless, the oxidation process is very complex and includes a cascade of reactions, many of which are simultaneous, giving rise to a large number of compounds of very different nature, which makes it very difficult to study them. Due to this, many efforts to monitor this process have been made, as well as to understand its mechanisms and develop strategies to avoid it.

To monitor oxidation, several classical parameters are used. Among these, the peroxide value (PV) and conjugated dienes (CD) are employed to evaluate the approximate concentration of the so-called primary oxidation compounds, and other parameters such as the anisidine value (AV) and TBARs index are used to evaluate some secondary oxidation compounds. However, although these parameters have been used for many years, the first parameters do not exclusively represent primary oxidation compounds, nor is the identity of the compounds monitored by the second parameters clear, as has been repeatedly commented [7,8,9,10], and this must be taken into account in the interpretation of their values. Other methods for estimating the oxidative stability of foods or of edible oils are based on the change in conductivity of an aqueous solution through which small volatile compounds released during oxidation provoked at high temperature pass, formic acid being one of the most representative. They are called oxidative stability index (OSI) methods, which employ accelerated oxidation conditions. The results of these methods are valid for comparisons between samples subjected to exactly the same conditions and refer to the formation of these small molecules, but cannot be extrapolated to that of other oxidation compounds. None of the methods mentioned above allow the identification of the different kinds of compounds formed in lipid oxidation, nor are they useful for providing information on oxidation mechanisms. They also have limitations for evaluating strategies aimed at avoiding oxidation processes in foods, as will be explained later.

In this context, edible oils and fats constitute a large group of foods, with a very varied composition in major and minor lipid components, of great nutritional and industrial interest, which can be taken as model systems for the study of the oxidation process. In addition to the determination of the classical parameters mentioned above, the study of oil oxidation can be approached addressing both the degradation of oil components and the formation of new components, following the evolution of both types of compounds throughout the oxidative process. Among these methods are those that involve the separation of the components of the complex mixtures formed in the oxidation process and the subsequent identification and quantification of the separated compounds. These methods have been applied mainly in the oxidation of pure compounds [11,12,13,14]. Others are based on different spectroscopic techniques that study these complex mixtures as a whole, without previous separation steps. This last group includes ^1^H NMR spectroscopy. Using this technique, great advances have been made since the first studies in which the estimation of the degradation of acyl fatty groups in the oxidation of edible oils was carried out [3,6,15,16]. Nowadays, this technique allows the estimation of the concentrations of any type of lipid component either coming from oxidation processes or not, as long as they have well-identified, non-overlapped ^1^H NMR signals [17,18]. Recently, taking into account ^1^H NMR spectral data of different oxidation compounds from previous studies [11,19,20,21,22,23,24,25,26,27,28,29], another important advance has been achieved in the study of the formation of a significant number of oxylipins generated in corn oil subjected to accelerated storage at 70 °C [30].

Due to the detrimental effects that lipid oxidation has on food and health, different strategies have been tested to prevent or hinder this degradative process. Among them, one that has been considered of great interest consists of adding compounds capable of acting as antioxidants to foods with the double objective of avoiding oxidation in the food and also endogenously after its ingestion.

However, like lipid oxidation, estimating the antioxidant capacity of a compound is a matter that requires much attention. For this purpose, several assays aimed at evaluating the ability of a compound, or a mixture of compounds, to scavenge free radicals (ABTS and DPPH methods) [31,32], to reduce ferric ions [33], or to absorb oxygen radicals (ORAC assay) [34] have been used to obtain information on its antioxidant capacity. Nevertheless, in these assays, neither the free radical to be scavenged nor the conditions under which the experiment is carried out are similar to those that occur in real systems. It is well known that, although these methods have been used in the past and are still currently in use, they do not always give concordant results. Other methods to evaluate the antioxidant ability of a compound involve its incorporation in a lipid matrix and the submission of the system to conditions under which the lipid can oxidize. In this case, the antioxidant capacity of a compound is usually estimated by determining some of the classic parameters mentioned above (PV, CD, AV, TBARs, OSI) after subjecting the lipid to oxidative conditions in the presence and absence of the compound object of study.

Among the lipid compounds to which antioxidant capacity has been attributed is alpha-tocopherol (αT), a well-known form of vitamin E present in many foods of vegetable and animal origin. It is perhaps for this attributed capability that the European legislation [35] allows edible oils to be enriched with this compound under the “*quantum satis*” principle, that is, without a set maximum limit. However, the antioxidant ability of this compound at any concentration and under any conditions is not clear.

Some studies on the ability of αT to inhibit the oxidation of pure compounds, such as linoleic acid dispersed in an aqueous media [36], or methyl linoleate in bulk [37] submitted to different oxidative conditions, have been carried out. In both cases, as the study was performed on pure compounds, the monitoring of the oxidation was made by high-performance liquid chromatography, estimating the concentration either of monohydroperoxy conjugated *Z*,*E*- and *E*,*E*-dienes in the first study or of monohydroperoxy, monohydroxy, and monoketo conjugated *Z*,*E*- and *E*,*E*-dienes in the second one. In the first study, it was concluded that αT, at a concentration of 3.8% by weight, exhibited pro-oxidant activity, whereas at lower concentrations (0.38% and 0.038% by weight), it exhibited antioxidant ability. In the second study, it was found that at concentrations ranging from 0.001% to 0.1% by weight, the formation of the aforementioned oxidation compounds was inhibited.

Likewise, the antioxidant ability of αT was also studied on much more complex substrates such as either purified [38] or almost free of tocopherols and sterols [39] soybean oil, submitted to different oxidative conditions. To evaluate the αT antioxidant or pro-oxidant ability in these two studies, classical methods such as peroxide values (PV) and oxygen headspace consumption in the first, and conjugated dienes (CD) in the second, were used. In both studies, the optimal concentration of αT to increase the oil oxidative stability was found to be 0.01% by weight, whereas this compound shows pro-oxidant activity at higher concentrations.

In addition, the effect of αT at various temperatures on the oxidation of complex substrates has also been addressed. Thus, the effect of several concentrations of αT dissolved in acetone, ranging from 0.02% to 0.22% by weight, on the oxidation of lard triglycerides, at several temperatures from 25 °C to 100 °C, was evaluated by means of peroxide values determination [40]. The results showed that αT is more effective in reducing the rate of lard triglycerides autoxidation as the temperature increases and the rate of lard triglycerides oxidation increased with increasing αT concentration, especially at low temperature.

Another study on purified olive oil, enriched with three different concentrations of αT, submitted to low temperatures (25 °C or 40 °C), has concluded, from parameters such as peroxide values and conjugated dienes, classically associated to primary oxidation compounds, that this compound under these conditions acts as a pro-oxidant [41]. From data of oxidative stability of the same samples determined by an OSI method that measures conductivity produced by secondary or further oxidation volatile compounds formed at 100 °C, it was concluded that αT at the same concentrations as above, at high temperature, acts as an antioxidant. However, the oxidation process is so complex that it could happen that under the same temperature, high or low, αT causes the generation of a higher concentration of primary oxidation compounds than in the reference oil sample and a lower concentration of those secondary or further oxidation volatile compounds responsible for producing the conductivity in the OSI method.

Another study very closely related to the above examined the oxidation of corn oil triglycerides, in the presence of various concentrations of αT, at 60 °C, for five days in the dark. Its results showed that at an αT concentration below 0.07% by weight, this compound inhibits the oxidation of these triglycerides, evaluated by peroxide value, and the opposite is true at higher concentrations [42]. However, the oxidative stability of the same corn oil triglycerides samples enriched in the same αT concentrations measured by the OSI method at 100 °C showed increased values with increased αT concentrations, although with diminishing effectiveness at concentrations higher than 0.07% by weight. Due to this, the authors concluded that αT activity can be classified as antioxidant or pro-oxidant depending on the method used for this evaluation. This conclusion also shows, as above, that the qualification of the behaviour of certain compounds such as αT as an antioxidant or pro-oxidant requires much more in-depth studies different to the simple determination of one or two parameters associated either with primary oxidation compounds or with oxidative stability, the latter measured from secondary or further oxidation compounds conductivity.

Taking into account all the aforementioned background, in this work, a real system, walnut oil with all its components, unenriched or enriched in two different concentrations of αT, as allowed by European legislation [35], will be subjected to storage at two different conditions, one of them at low-temperature accelerated storage (40 °C), and the other one at intermediate-temperature accelerated storage (70 °C), will be studied by ^1^H NMR spectroscopy until very advanced stages of its oxidation process. The aim of the study is to analyze the influence of both the degree of enrichment in αT of the oil and the storage temperature on the oxidative stability of this polyunsaturated oil rich in omega-6 acyl groups, which means elucidating the αT behaviour as either an antioxidant or pro-oxidant under these conditions. This elucidation will not be based on the determination of one or two parameters associated with certain oxidation compounds, as has been carried out in previous studies, but on the identification and quantification throughout the oxidation process of all kinds of compounds that can be studied by ^1^H NMR. This study will require the identification of an important number of oxidation compounds, many of them identified recently for the first time in edible oil oxidation, reinforcing in this way a previous study, and taking a step forward [30]. In addition, it will require the estimation of their concentration along the oxidation process by using the same method in all cases without chemical modification of the sample or previous separation steps. In this way, this study could contribute to shedding light on the controversy found in previous studies [42] regarding the dependence of the qualification of αT behaviour as an antioxidant or pro-oxidant depending on the method of study used. The interest of this work is great due to the fact that the oxidation compounds subject to study, in addition to being formed in food autoxidation, can also be formed endogenously during food digestion and even in cells and tissues.

## 2. Materials and Methods

### 2.1. Subject Samples of Study

The subject samples of study are walnut oil (W), purchased in a local supermarket, and this same oil enriched in alpha-tocopherol (αT), in proportions of 0.2% by weight (W_02_) and of 2% by weight (W_2_). The composition of walnut oil in fatty acyl groups was estimated by ^1^H NMR, as in previous works [43,44,45]. Their molar percentages are 11.5 ± 0.1% of linolenic, 62.6 ± 0.5% of linoleic, 14.0 ± 1.2% of oleic and 11.8 ± 1.2% of saturated acyl groups. It must be mentioned that, as is usual in walnut oils, the natural content of αT in this walnut oil is very low, not detectable by ^1^H NMR. The αT used to enrich the walnut oil was purchased from Sigma-Aldrich (St. Louis, MO, USA) and had a purity of 98.2%.

### 2.2. Storage Conditions

Aliquots of 10 g of walnut oil alone (sample W) and enriched either with 0.2% or with 2% by weight of αT (samples W_02_, or W_2_, respectively) were poured into glass Petri dishes of 80 mm diameter. These were kept in a convection oven either at 40 °C or at 70 °C in the presence of air until advanced stages of their oxidation process, trying to simulate accelerated storage conditions at low temperature in the first case and at intermediate temperature in the second. In both cases, the experiment was carried out by duplicate. The evolution of each sample under the aforementioned conditions was monitored by using ^1^H NMR spectroscopy.

### 2.3. Study of the Samples Evolution by ^1^H NMR Spectroscopy

#### 2.3.1. ^1^H NMR Spectroscopy Operating Conditions

The ^1^H NMR spectra of several samples, W, W_02_, and W_2_, throughout the storage at both temperatures, were acquired in duplicate by using a Bruker Avance 400 spectrometer (Bruker, Billerica, MA, USA) operating at 400 MHz. To this aim, approximately 175 µL of the above-mentioned samples were mixed in a 5 mm-diameter tube with 425 µL of deuterated chloroform that contained 0.2% of non-deuterated chloroform and 0.03% of tetramethylsilane (TMS) as an internal reference (Cortec, Paris, France). The acquisition parameters were the same as those used in previous studies [15,17]: spectral width 5000 Hz, relaxation delay 3 s, number of scans 64, acquisition time 3.744 s and pulse width 90°, with a total acquisition time of 8 min 55 s. The relaxation delays and acquisition times selected allow the complete relaxation of the protons, the signal areas thus being proportional to the number of protons that generate them, making their use for quantitative purposes possible. The experiments were carried out at 25 °C. The ^1^H NMR spectra were plotted at a fixed value of absolute intensity to be valid for comparative purposes using the MestreNova program (Mestrelab Research, Santiago de Compostela, Spain).

#### 2.3.2. Identification of Components

The identification of components present in the original oil as well as of those generated in the oxidation processes was carried out on the basis of the assignment of the ^1^H NMR signals to the different kinds of hydrogen atoms of the different structures. These signals, their chemical shifts and their assignments to the several hydrogen atoms are given in Tables S1–S16 (Supplementary Material). These assignments were made taking into account previous studies, as indicated in each table, or on the basis of the signals of standard compounds acquired for this study. The latter include: 2*E*-hexenal, 2*E*-heptenal, 2*E*-decenal, 2*E*,4*E*-hexadienal, 2*E*,4*E*-heptadienal, 2*E*,4*E*-decadienal, 4,5-epoxy-2*E*-decenal, 12,13-epoxy-9*Z*-octadecenoic acid methyl ester (isoleukotoxin methyl ester), 2-pentylfuran, 2-ethylfuran, amylformate, and octylformate acquired from Sigma-Aldrich (St. Louis, MO, USA); 9,10-epoxy-12*Z*-octadecenoic acid (leukotoxin), 12,13-*E*-epoxy-9*Z*-octadecenoic acid, 4-hydroxy-2*E*-nonenal, 4-hydroperoxy-2*E*-nonenal, 4-oxo-2*E*-nonenal, 9,10-dihydroxy-12*Z*-octadecenoic acid (leukotoxin diol), 12,13-dihydroxy-9*Z*-octadecenoic acid (isoleukotoxin diol), 9-keto-12,13-*E*-epoxy-10*E*-octadecenoic acid, 9-keto-10*E*,12*E*-octadecadienoic acid, 9-keto-10*E*,12*Z*-octadecadienoic acid, 13-keto-9*Z*,11*E*-octadecadienoic acid and 12*R*-hydroxy-9*Z*-octadecenoic acid methyl ester (ricinoleic acid methyl ester), purchased from Cayman Chemical (Ann Arbor, MI, USA); 9*S*-hydroxy-10*E*,12*E*-octadecadienoic acid (Dimorphecolic acid), methyl 13*S*-hydroxy-9*S*,10*R*-epoxy-11*E*-octadecenoate, 11*S*-hydroxy-9*S*,10*S*-epoxy-12*Z*-octadecenoic acid methyl ester, 13*S*-hydroxy-11*S*,12*S*-epoxy-9*Z*-octadecenoic acid methyl ester, 9-hydroxy-10-keto-12*Z*-octadecenoic acid, 9,10-*Z*-12,13-*Z*-diepoxyoctadecanoic acid and 13-hydroxy-12-keto-9*Z*-octadecenoic acid acquired from Larodan (Malmö, Sweden).

#### 2.3.3. Quantification of the Components

The estimation of the concentration of the different functional groups or groups of compounds present in the several samples, throughout the storage, was possible because the area of each ^1^H NMR spectral signal is proportional to the number of protons that generates it, and this proportionality is constant for all kinds of proton. Taking this into account, the estimation of the concentration of the polyunsaturated fatty acyl groups, of the several derivatives, and of αT in relation to that of triglycerides (TG) was carried out throughout the storage. For this, the area of the signal of the characteristic protons of the functional groups and that of the protons of the triglycerides were used. Due to the low level of hydrolysis that occurred during oil storage, triglycerides (TG) can be considered as an internal reference. Taking into account all of the above, the equation used to estimate the concentrations of the different derivatives was:

[X] = [(A_X_/n)/(A_TG_/4)] × 1000, (1)

where “A_X_” is the area of the signal selected for the quantification of the X functional group, “n” is the number of protons that generate this signal and “A_TG_” is the area of the protons at *sn*-1 and *sn*-3 positions in the triglyceride backbone of TG (signal H in Table S1, see Supplementary Material). The area of the signals was determined by using the equipment software and the integrations were performed three times to obtain average values. The concentration thus obtained is expressed in millimole per mol of triglyceride (mmol/mol TG). It is worth noting that, in some cases, the signals of certain compounds overlap with the signals of other compounds. In these cases, the corresponding corrections have to be applied in order to avoid underestimations or overestimations of the concentration of the functional group or derivative.

### 2.4. Statistical Analysis

The data represented in the different figures are average values of at least two determinations and those given in tables come from the aforementioned average values. Microsoft Office Excel 2016 (One Microsoft Way, Redmond, WA, USA) was used to carry out the statistical analysis and for the graphical representation of the obtained values.

## 3. Results

As mentioned before, walnut oil samples with different alpha-tocopherol (αT) enrichment degrees, W, W_02_, and W_2_, were stored at two different temperatures, 40 °C and 70 °C, in the presence of air, and their evolution was followed by means of ^1^H NMR up to advanced stages of their oxidation process. Firstly, the attention was focused on the evolution of the most unsaturated acyl groups, that is to say, on the evolution of their linoleic and linolenic acyl groups.

### 3.1. Evolution of Polyunsaturated Acyl Groups Concentration. Influence of Both Storage Temperature and Enrichment Degree of the Oil in αT

**Figure 1:**
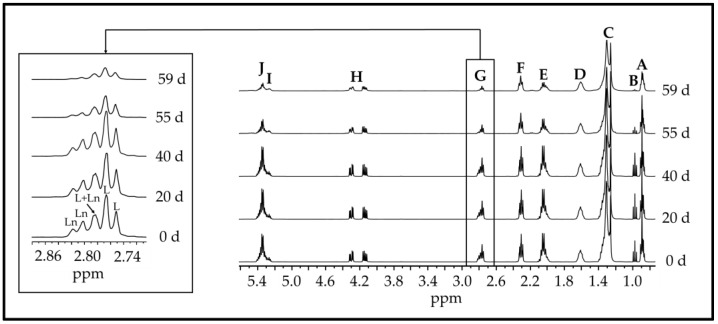
^1^H NMR spectra of the walnut oil (W) at different days (d) of its accelerated storage at 40 °C, and the enlargement of signal G due to the bis-allylic methylene protons of their linoleic and linolenic acyl groups. As can be observed in this figure, the intensity of some of the walnut oil spectral signals (signal assignments in Table S1) decreases through time due to the degradation of the most unsaturated acyl groups. Indeed, some of them had almost disappeared at the most advanced stage of the oxidation process. This behaviour is also observed, in general terms, in the spectra of the other samples submitted either at 40 °C or at 70 °C. However, both factors, the αT enrichment degree and storage temperature, have an important influence on the evolution of the polyunsaturated acyl group concentration in this oil.

It is well known that under accelerated storage conditions, the degradation of the acyl groups of oils is produced [30,46,47], the most unsaturated ones being the most affected. This degradation can be directly observed in the ^1^H NMR spectra of the oil samples submitted to storage. As an example, Figure 1 shows the ^1^H NMR spectra of walnut oil sample (W) submitted to 40 °C at certain time-points of its degradation process.

**Figure 2:**
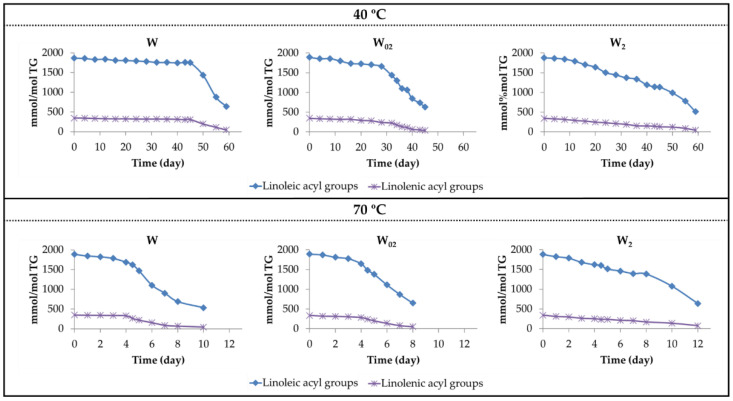
Evolution of the concentration, expressed as mmol/mol TG, of linoleic and linolenic acyl groups in walnut oil with different enrichment levels of αT (W, W_02_, and W_2_), subjected either to 40 °C or to 70 °C up to advanced stages of the oxidation process.

The concentration of linoleic and linolenic acyl groups in relation to TG concentration throughout the storage was estimated from the area of their bis-allylic protons [45], using the equation described in Section 2.3.3. The results obtained for the different oil samples are represented in Figure 2.

As shown in Figure 2, the evolution of the concentration of both acyl groups follows a linear path with two slopes in most cases, the first being smaller than the second. The slope of these lines in each stage represents the degradation rate of the corresponding acyl group. These are given in Table 1.

From the data in Table 1 and Figure 2, it is clear that αT enrichment, at any temperature, causes an increase in the degradation rate of the acyl groups in the first stage and a decrease in the second stage, relative to those of the non-enriched sample. Because of this, the differences in the degradation rates of both stages in the same sample decrease as the αT enrichment increases, and consequently, in the samples with the highest αT enrichment degree, the degradation rate tends to be the same in both stages for linolenic group.

Increasing the storage temperature increases the degradation rate at both degrees of αT enrichment, so that the differences between 40 °C and 70 °C are very noticeable, significantly affecting the total duration of the degradation process, as shown in Table 1 and Figure 2. Although increasing the temperature causes an increase in the degradation rate at both stages in all samples, it does not modify the trend caused by αT enrichment. The results obtained at 70 °C are in agreement with those previously reported by Martin-Rubio et al. [48,49] when studying the oxidation process of soybean oil enriched with different levels of αT submitted to storage at 70 °C.

The results obtained provide evidence that the walnut oil enrichment in αT in the assayed concentrations clearly reduces the oil oxidative stability at both temperatures, this reduction being greater the higher the αT enrichment, showing that αT acts as a pro-oxidant. Likewise, the higher the storage temperature, the lower the oxidative stability of the walnut oil, as is already known.

### 3.2. Formation and Evolution of the Concentration of Oxidation Products. Influence of Both Storage Temperature and Enrichment Degree of the Oil in αT

The nature and concentration of the oxidation compounds formed in the different oil samples throughout the storage will be dependent not only of the oil composition but also on its αT enrichment level and on the storage conditions.

#### 3.2.1. Primary Oxidation Compounds. Long Chains Supporting Hydroperoxy Groups and Conjugated *Z*,*E*- or *E*,*E*-dienes

It is known that in the oxidation process of lipids, the first compounds formed are monohydroperoxides; for this reason, they are named primary oxidation compounds. The concentrations of these increase as the oxidation advances to reach a maximum, after which it diminishes sharply due to their degradation to give rise to the formation of secondary or further oxidation compounds.

**Figure 3:**
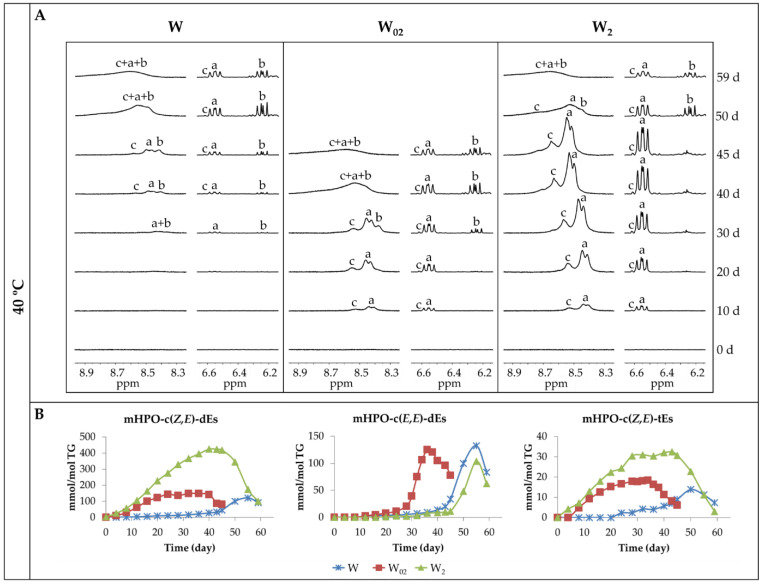
(**A**) ^1^H NMR regions of the spectra of walnut oil samples with different αT enrichment degrees (W, W_02_ and W_2_), at different days (d) of their accelerated storage at 40 °C, in which signals of hydroperoxy protons in chains supporting conjugated dienic systems derived from linoleic and linolenic appear. Signals a and b are due to protons of mHPO-c(*Z*,*E*)-dEs and mHPO-c(*E*,*E*)-dEs respectively, derived from linoleic acyl group. Signals c are due to protons of mHPO-c(*Z*,*E*)-tEs derived from linolenic acyl group. (**B**) Evolution of the concentration of each kind of hydroperoxides throughout the storage up to advanced stages of the oxidation process.

Figure 3A shows some ^1^H NMR regions of walnut oil spectra enriched with different concentrations of αT throughout their storage at 40 °C. In these regions, some signals of the compounds formed in the oxidation of this oil appear. Thus, in the region between 8.20 ppm and 8.90 ppm, the signals of the protons of hydroperoxy groups associated to conjugated *Z*,*E-* and *E*,*E*-dienic systems (mHPO-c(*Z*,*E*)-dEs and mHPO-c(*E*,*E*)-dEs), (signals a and b, respectively) derived from the linoleic group are the first ones becoming visible, in agreement with previous studies [50,51]. Furthermore, in the same region, another signal (signal c) appears due to the protons of the hydroperoxy groups supported on triene chains with two of the double bonds forming a conjugated *Z*,*E*-dienic system (mHPO-c(*Z*,*E*)-tEs), the latter being hydroperoxides derived from the linolenic group.

In addition, in Figure 3A, the ^1^H NMR region between 6.20 ppm and 6.65 ppm of the same spectra is also shown. In it, some of the signals of the protons of the conjugated dienic systems mentioned above (named with the same letters a, b and c) appear, in agreement with previous studies [50,51,52], the assignments of which are given in Tables S2 and S3. It is worth mentioning that the multiplet signal of the protons of the c(*Z*,*E*)-dienic system of the hydroperoxides derived from linoleic group (signal a) partially overlaps with that of the same kinds of conjugated dienic system protons derived from linolenic group (signal c), the reason for which being that the latter signal is only partially visible.

From the observation of these signals, it is evident that the first compounds formed during storage are monohydroperoxides conjugated *Z*,*E*-dienic systems (mHPO-c(*Z*,*E*)-dEs and mHPO-c(*Z*,*E*)-tEs), coming from both omega-6 and omega-3 acyl groups, respectively (signals a and c). In addition, the formation of these kinds of compounds occurs earlier the higher the enrichment degree in αT is. Likewise, it is also evident that the formation of monohydroperoxides with conjugated *E*,*E*-dienic systems (mHPO-c(*E*,*E*)-dEs) derived from linoleic group (signal b) is delayed in relation to that of the monohydroperoxides with conjugated *Z*,*E*-dienic systems (mHPO-c(*Z*,*E*)-dEs) to a greater extent the higher the enrichment in αT is. However, the formation of both kinds of hydroperoxides occurs almost simultaneously in the walnut oil sample non-enriched in αT.

As indicated in the experimental section, from the area of the conjugated dienic proton signals, the concentration of the aforementioned hydroperoxides in the different oil samples during storage at 40 °C was estimated by using the area of the proton signals in the region between 6.2 and 6.6 ppm and plotted versus time in Figure 3B. It can be observed that, in accordance with the above, the higher the enrichment level of the oil in αT, the earlier the formation of hydroperoxy conjugated *Z*,*E*-dienes coming from both linoleic (mHPO-c(*Z*,*E*)-dEs) and linolenic (mHPO-c(*Z*,*E*)-tEs), and the opposite occurs with hydroperoxy conjugated *E*,*E*-dienes coming from linoleic (mHPO-c(*E*,*E*)-dEs). Furthermore, it is noticeable that the concentration of both kinds of *Z*,*E*-isomers, either coming from linoleic or from linolenic, increases when increasing the degree of enrichment in αT.

These results suggest that the presence of αT not only accelerates the formation of hydroperoxy conjugated *Z*,*E*-dienes derived from the two above-cited acyl groups but also increases their stability retarding their degradation, which could explain their increase in concentration, in agreement with some previous studies [36,53,54,55]. Simultaneously a great delay is produced in the formation of the hydroperoxy conjugated *E*,*E*-dienes, occurring when the concentration of their *Z*,*E*-counterparts has diminished significantly due to their degradation. The inhibitory effect of αT delaying the isomerization from *Z*,*E* to *E*,*E* has also been observed in previous studies [36,37,48,49,55,56,57]. Some of these studies have attributed this inhibitory effect to the strong H-donating capacity of αT to the peroxyl radical, which could inhibit the rearrangement of hydroperoxy conjugated *Z*,*E*-dienes to the more thermodynamically stable hydroperoxy conjugated *E*,*E*-dienes.

**Figure 4:**
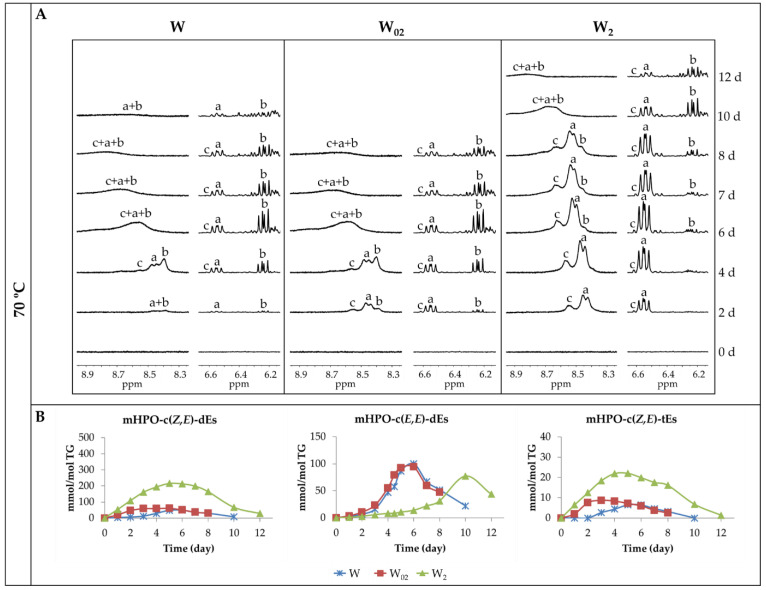
(**A**) ^1^H NMR regions of the spectra of walnut oil samples with different αT enrichment degrees (W, W_02_ and W_2_), at different days (d) of their accelerated storage at 70 °C, in which signals of hydroperoxy protons in chains supporting conjugated dienic systems derived from linoleic and linolenic appear. Signals a and b are due to protons of mHPO-c(*Z*,*E*)-dEs and mHPO-c(*E*,*E*)-dEs respectively, derived from linoleic acyl group. Signals c are due to protons of mHPO-c(*Z*,*E*)-tEs derived from linolenic acyl group. (**B**) Evolution of the concentration of each kind of hydroperoxides throughout the storage up to advanced stages of the oxidation process.

Figure 4A shows the same spectral regions as those shown in Figure 3A of the ^1^H NMR spectra of the same walnut oil samples W, W_02_ and W_2_ throughout their storage at 70 °C in the presence of air. It can be observed that in the regions between 8.20 and 8.90 ppm and between 6.20 and 6.65 ppm, the same signals (a, b and c) present in the spectra of the samples submitted at 40 °C appear. The appearance of these signals occurs much earlier in the experiment at 70 °C than at 40 °C. However, the effect that the enrichment of walnut oil with αT provokes the evolution of the oxidation process during storage at 70 °C is in line with that observed in the oxidation process during storage at 40 °C. From these results, it is also clear that in the oxidation process of walnut oil at 70 °C, the higher the degree of αT enrichment, the earlier the formation of hydroperoxy conjugated *Z*,*E*-dienes derived from both linoleic and linolenic groups and the later the formation of hydroperoxy conjugated *E*,*E*-dienes derived from linoleic groups. However, the rate of both formation and degradation of all types of hydroperoxides occurs much more rapidly at 70 °C than at 40 °C, while maintaining the effect caused by the presence of αT. In fact, as shown in Figure 3B and Figure 4B, the total oxidation process at 70 °C takes between 8 and 12 days, while at 40 °C, it takes between 45 and 59 days.

In addition to the abovementioned, it should be highlighted that in the oxidation process at 70 °C (see Figure 4B), the maximum concentration reached by all kinds of hydroperoxides in all samples is much smaller than that reached at 40 °C (see Figure 3B). This could be due to a lower stability of these intermediate oxidation compounds at high rather than at low temperature. It is also noticeable that, at 70 °C, both in the non-enriched sample and in the sample with the lowest αT enrichment degree, the concentration of hydroperoxy conjugated *E*,*E*-dienes is higher than that of their *Z*,*E*-counterparts coming from linoleic, during a variable period of time. This can be explained because the isomerization from *Z*,*E* to *E*,*E* is favoured by the temperature increase since the latter isomers are more stable thermodynamically than the former [57,58,59].

The results obtained show that the enrichment of walnut oil with αT reduces its oxidative stability under the conditions of this study at both temperatures, since the presence of αT to a greater extent accelerates the formation of hydroperoxy conjugated *Z*,*E*-dienes the higher the degree of enrichment [10,38,48,49]. This acceleration in the formation of hydroperoxy conjugated *Z*,*E*-dienes by the presence of αT could be considered as a pro-oxidant effect of this compound and has been attributed to the possible participation of the tocopheryl radical in different propagative reactions [60]. Furthermore, it is also evident that αT enrichment increases the stability of the formed hydroperoxy conjugated *Z*,*E*-dienes, so that they reach higher concentrations in the more enriched samples. Moreover, as already mentioned, αT hinders the isomerisation of linoleic-derived hydroperoxy conjugated *Z*,*E*-dienes to the corresponding more thermodynamically stable *E*,*E*-counterparts, delaying the formation of the latter hydroperoxides with respect to the former, even at 70 °C.

It is worth noting in all samples in the first oxidation stage, the parallelism between the degradation rates of linoleic and linolenic acyl groups, shown in Figure 2 and given in Table 1, and the rate of concentration increase of primary oxidation compounds, represented mainly by that of hydroperoxy conjugated *Z*,*E*-dienes (see Figure 3B and Figure 4B). The large difference in the concentration of *Z*,*E*-hydroperoxides derived from linoleic and linolenic groups achieved in the different walnut oil samples subjected to storage, shown in Figure 3B and Figure 4B, is due to the former acyl group in walnut oil being in a much higher concentration than the latter.

**Figure 5:**
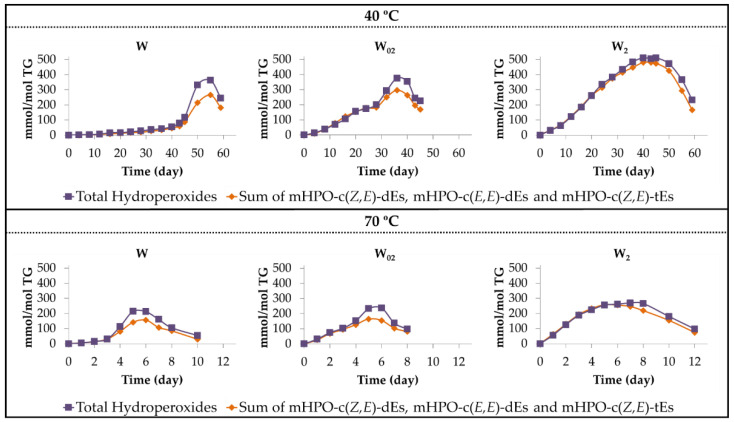
Evolution of the concentration of both primary oxidation compounds and total hydroperoxy groups, in the different walnut oil samples throughout the storage, at either 40 °C or 70 °C, up to advanced stages of the oxidation process.

Finally, it only remains to add that the total area of the ^1^H NMR signals of the protons of the hydroperoxy groups match, during an initial period of time, with the sum of the area of the signals of the hydroperoxy conjugated *Z*,*E*- and *E*,*E*-dienic protons, as shown in Figure 5. This means that the only hydroperoxy groups present in the oil during an initial period of time are of the aforementioned hydroperoxy conjugated dienes.

However, after a certain time under storage conditions, as Figure 5 shows, the concentration of total hydroperoxy groups is higher than the sum of the concentrations of primary oxidation compounds (mHPO-c(*Z*,*E*)-dEs, mHPO-c(*Z*,*E*)-tEs and mHPO-c(*E*,*E*)-dEs), suggesting that the formation of secondary oxidation compounds also having hydroperoxy groups has taken place.

#### 3.2.2. Long-Chain Secondary Oxidation Compounds Supporting Hydroperoxy Groups

In a previous study on the oxidation undergone by corn oil submitted to storage conditions at 70 °C, the formation of secondary oxidation compounds supporting hydroperoxy groups was observed [30]. For this reason, it could be expected that these compounds could also be formed in the samples in this study and that their formation could be affected by both the αT enrichment level of walnut oil and the storage temperature. The detailed observation of the ^1^H NMR spectra of these samples permitted the detection of signals attributable to dihydroperoxy non-conjugated dienes (dHPO-nc-dEs) and non-vicinal monohydroperoxy monoepoxy monoenes (non-vicinal mHPO-mEPO-mEs).

**Figure 6:**
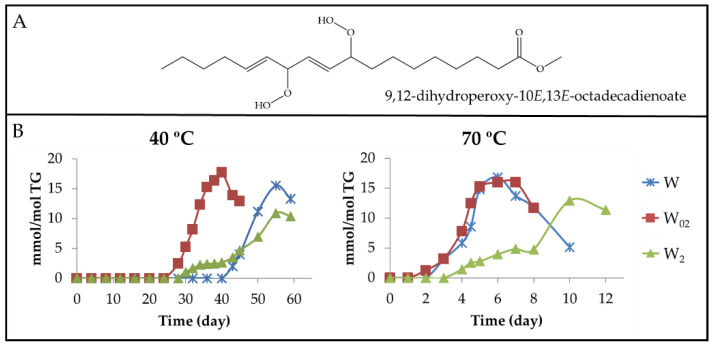
(**A**) Structure of 9,12-dihydroperoxy-10*E*,13*E*-octadecadienoate as an example of a dHPO-nc(*E*,*E*)-dE. (**B**) Evolution of the concentration, expressed as mmol/mol TG, of dHPO-nc(*E*,*E*)-dEs in walnut oil samples with different enrichment levels of αT (W, W_02_ and W_2_), subjected to either 40 °C or 70 °C, up to advanced stages of the oxidation process.

(a) Dihydroperoxy non-conjugated *E*,*E*-dienes (dHPO-nc(*E*,*E*)-dEs). The formation of these compounds is evidenced by the appearance of a multiplet signal centred near 4.82 ppm (Table S4) in the ^1^H NMR spectra of the different walnut oil samples. This signal is attributable to the carbinol methine protons of the hydroperoxy groups of 9,12-dihydroperoxy-10*E*,13*E*-octadecadienoate (see Figure 6A) and/or of 10,13-dihydroperoxy-8*E*,11*E*-octadecadienoate [61]. Furthermore, the spectra of these compounds have other signals common with those present in the spectra of other oxidation compounds (see Table S4). The integration of the area of the first cited signal allows one to estimate the evolution of the concentration of dHPO-nc(*E*,*E*)-dEs in the samples throughout the storage time. These data are depicted versus the storage time in Figure 6B.

Data represented in Figure 3B, Figure 4B and Figure 6B suggest that the formation of these compounds begins, at both temperatures in the samples, when the concentration of their precursor mHPO-c(*Z*,*E*)-dEs [62] reaches a certain level and always after the mHPO-c(*E*,*E*)-dEs formation. The concentration of the precursor at the onset of the formation of dHPO-nc(*E*,*E*)-dEs is different in each sample, being greater the higher the enrichment in αT and smaller the higher the storage temperature; that is to say, it depends on both enrichment degree in αT and storage temperature.

Two factors appear to govern the rate of formation of these compounds, namely the stability of their precursor in each sample and the difficulty of isomerization from *Z*,*E* to *E*,*E* possibly required for their formation. Both factors are directly related to the αT concentration in the oil and inversely related to the storage temperature. It is well known that the increase in temperature facilitates isomerization from *Z*,*E* to *E*,*E* because the latter isomers are more thermodynamically stable than the first. It seems that the previously mentioned factors monitor not only the time at which the formation of these compounds begins in each sample but also their rate of formation and the maximum concentration that they reach. The different inhibition capacity for the isomerisation from *Z*,*E* to *E*,*E* in the various samples, due to their different degree of enrichment in αT, is a decisive factor for the formation of these compounds and in fact dHPO-nc(*E*,*E*)-dEs reach their maximum concentration at the same time as mHPO-c(*E*,*E*)-dEs.

Finally, it only remains to be added that the evolution of the concentration of these compounds proves their role as intermediate compounds because their concentration reaches a maximum, indicating that after this time point, their rate of formation is lower than that of their degradation. In agreement with this, previous studies have described that dHPO-nc(*E*,*E*)-dEs are intermediate compounds in the formation of aldehydes [27,57,62,63].

**Figure 7:**
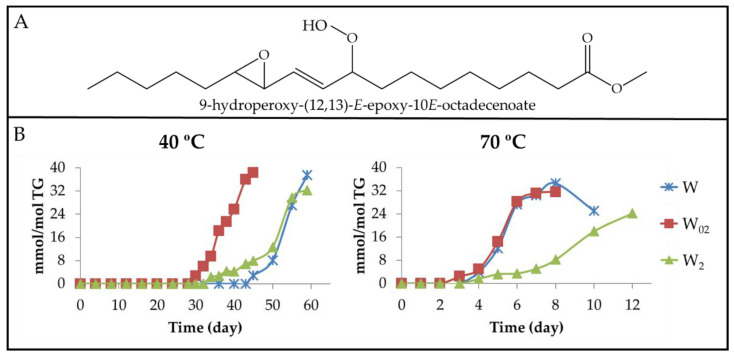
(**A**) Structure of 9-hydroperoxy-(12,13)-*E*-epoxy-10*E*-octadecenoate as an example of non-vicinal mHPO-mEPO-*E*-mE. (**B**) Evolution of the concentration expressed as mmol/mol TG, of all non-vicinal mHPO-mEPO-*E*-mEs present in walnut oil samples with different enrichment levels of αT (W, W_02_ and W_2_), submitted to either 40 °C or 70 °C, up to advanced stages of the oxidation process.

(b) Non-vicinal monohydroperoxy monoepoxy *E*-monoenes (non-vicinal mHPO-mEPO-*E*-mEs). It has been described that mHPO-c(*Z*,*E*)-dEs are also the precursors of these compounds. Thus, 13-HPO-c(11*E*,9*Z*)-dE is considered the precursor of both 9-HPO-(12,13)-*E*-EPO-10*E*-mE (see Figure 7A) [20] and 13-HPO-(9,10)-*Z*-EPO-11*E*-mE [64]. The formation of the first involves an epoxidation and the formation of an *E*-double bond, whereas that of the second could occur by a simple epoxidation of the *Z*-double bond. In the same way, it could be expected that the oxidation of 9-HPO-c(10*E*,12*Z*)-dE could yield 13-HPO-(9,10)-*E*-EPO-11*E*-mE or to 9-HPO-(12,13)-*Z*-EPO-10*E*-mE. All these compounds show, in their ^1^H NMR spectra, a clear double doublet signal centred near 5.85 ppm (see Table S5), which is visible in the spectra of all walnut oil samples after a certain storage time. Furthermore, the spectra of these non-vicinal mHPO-mEPO-*E*-mEs have other signals common with those of other oxidation compounds (see Table S5). From the area of the signal mentioned above, the concentration of all non-vicinal mHPO-mEPO-*E*-mEs has been estimated jointly throughout the storage time and has been depicted in Figure 7B.

The observation of Figure 6B and Figure 7B proves that the formation of these compounds always starts after that of mHPO-c(*E*,*E*)-dEs, and only slightly later than that of dHPO-nc(*E*,*E*)-dEs, although they are generated in higher concentrations than the latter. The parallelism in the formation of non-vicinal mHPO-mEPO-*E*-mEs and dHPO-nc(*E*,*E*)-dEs suggests that the same factors influence and govern their formation. The role of non-vicinal mHPO-*E*-mEPO-*E*-mEs as intermediate compounds in the formation of 4,5-EPO-2*E*-alkenals [65] is also noteworthy.

Finally, it is noteworthy that there is a strong temporal agreement between the onset of formation of dHPO-nc(*E*,*E*)-dEs and non-vicinal mHPO-mEPO-mEs (see Figure 6B and Figure 7B), and the time at which the concentration of total hydroperoxides and that of the sum of primary oxidation compounds becomes different (See Figure 5). Likewise, these figures also show that there is a good agreement between the time at which this difference reaches the highest value and the time at which the concentration of these two kinds of compounds reaches the maximum value. There is also agreement in that the smallest differences between the concentration of total hydroperoxides and that of primary oxidation compounds occurs in the oil samples with the highest αT enrichment degree at both temperatures, which are those generating the lowest concentration of dHPO-nc(*E*,*E*)-dEs and non-vicinal mHPO-mEPO-*E*-mEs. These agreements reinforce both the obtained results and the interpretations made.

#### 3.2.3. Long-Chain Secondary or Further Oxidation Compounds Supporting a Hydroxy Group

The formation of monohydroxy conjugated dienic derivatives as well as that of non-vicinal monohydroxy monoepoxy derivatives has been previously reported in the oxidation of polyunsaturated oils [30,48,49]. Therefore, it could be thought that both types of compounds could also be formed in the samples under study here, and it is to be expected that both the storage temperature and the level of αT enrichment affect their formation.

**Figure 8:**
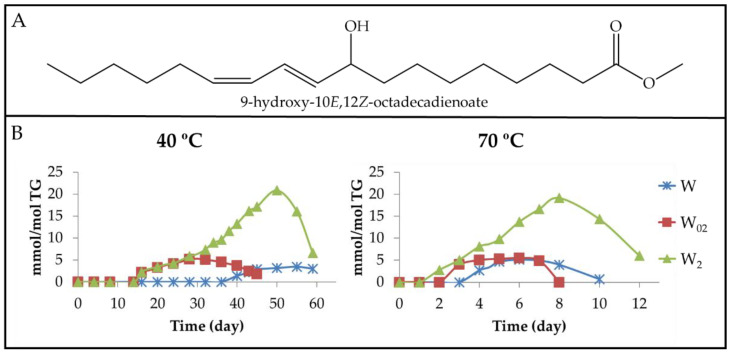
(**A**) Structure of 9-hydroxy-10*E*,12*Z*-octadecadienoate as an example of mHO-c(*Z*,*E*)-dE. (**B**) Evolution of the concentration expressed as mmol/mol TG, of mHO-c(*Z*,*E*)-dEs in walnut oil with different enrichment levels of αT (W, W_02_ and W_2_), submitted to either 40 °C or 70 °C up to advanced stages of the oxidation process.

(a) Monohydroxy conjugated dienes (mHO-c-dEs) give characteristic ^1^H NMR signals (see Table S6) in the region of conjugated dienic protons, the reason for which being that their detection and quantification could be carried out. In the samples studied here, only mHO-c(*Z*,*E*)-dEs (see Figure 8A) have been detected and the evolution of their concentration throughout the storage has been represented in Figure 8B.

The onset of formation of these derivatives occurs when the concentration of the precursors, which are mHPO-c(*Z*,*E*)-dEs [26], has reached a certain level, which is different for each sample, being higher the greater the degree of enrichment of walnut oil in αT. This occurs at both temperatures, but the concentration of the precursor at the onset formation of these compounds is smaller at 70 °C than at 40 °C. Furthermore, the highest concentration of mHO-c(*Z*,*E*)-dEs is reached in the sample most enriched in αT at both temperatures, being somewhat higher at 40 °C than at 70 °C. In summary, the formation of these derivatives begins earlier and they reach higher concentrations in the oil samples enriched in αT than in the non-enriched sample. The role of these compounds as intermediate compounds is evidenced because in all cases, their concentration increases until it reaches a maximum, after which it decreases. In fact, the role of these kinds of compounds as precursors in the formation of 4-HO-2*E*-alkenals has been described [66].

The results obtained here are in agreement with the ones described in a previous paper of soybean oil enriched with αT submitted to 70 °C, where higher concentrations of mHO-c-dEs were detected the higher the concentration of αT was [48,49]. Likewise, these results are also in agreement with those obtained from methyl linoleate *Z*,*E*-hydroperoxides [55].

**Figure 9:**
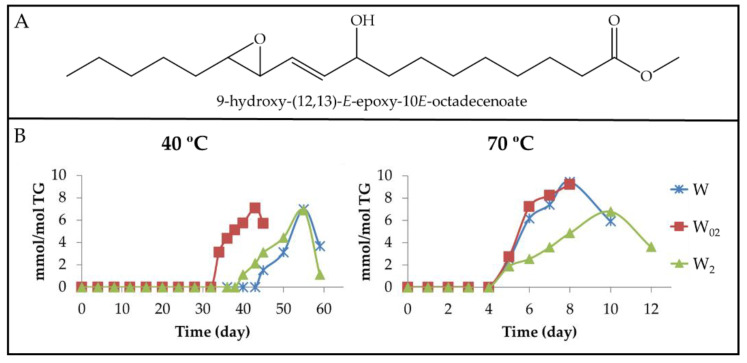
(**A**) Structure of 9-hydroxy-(12,13)-*E*-epoxy-10*E*-octadecenoate as an example of non-vicinal mHO-mEPO-*E*-mE. (**B**) Evolution of the concentration expressed as mmol/mol TG, of the non-vicinal mHO-mEPO-*E*-mEs in walnut oil with different enrichment levels in αT (W, W_02_ and W_2_), submitted to either 40 °C or 70 °C up to advanced stages of the oxidation process.

(b) Non-vicinal monohydroxy monoepoxy *E*-monoenes (non-vicinal mHO-mEPO-E-mEs). These compounds could be formed from mHPO-c(*Z*,*E*)-dEs [20,67], or they can be generated either from mHO-c(*Z*,*E*)-dEs or from non-vicinal mHPO-mEPO-*E*-mEs, both derived from the first one. Their concentration in the sample can be expected to be very low, taking into account the concentration of some of their precursors. In the ^1^H NMR spectra of the samples under study, signals attributable to 9-HO-(12,13)-*E*-EPO-10*E*-mE (structure shown in Figure 9A) and/or 13-HO-(9,10)-*E*-EPO-11*E*-mE on one side or to 13-HO-(9,10)-*Z*-EPO-11*E*-mE and/or 9-HO-(12,13)-*Z*-EPO-10*E*-mE on the other appear. All these non-vicinal mHO-mEPO-*E*-mEs have protons giving a common double doublet spectral signal centred at 5.94–5.95 ppm (see Table S7), which has very low intensity, and other signals that are common with those of other compounds such as non-vicinal mHPO-mEPO-*E*-mEs (see Tables S5 and S7). From the area of the signal previously mentioned, the concentration of all non-vicinal mHO-mEPO-*E*-mEs was estimated jointly in the samples, and its evolution throughout the storage time is represented in Figure 9B.

It is noteworthy, as Figure 6B, Figure 7B and Figure 9B show, that the formation path of non-vicinal mHO-mEPO-*E*-mEs is similar to that of dHPO-nc(*E*,*E*)-dEs and non-vicinal mHPO-mEPO-*E*-mEs in the same samples, and this suggests that the factors that govern the formation of all of them are the same. These can be resumed in the stability of the precursor and the inhibitory power of the system for the isomerization from *Z*,*E* to *E*,*E*, both factors being directly related to the concentration of αT.

#### 3.2.4. Long-Chain Secondary or Further Oxidation Compounds Supporting a Keto Group

Oxidation derivatives such as monoketo conjugated dienes (mKO-c-dEs), non-vicinal and vicinal monoketo monoepoxy monoenes (non-vicinal and vicinal mKO-mEPO-mEs) and non-vicinal monoketo monohydroxy monoenes (non-vicinal mKO-mHO-mEs) have been described as being formed in the oxidation of oils rich in the linoleic acyl group [30,48,49]. For this reason, it is to be expected that they could also be formed in the oxidation undergone by walnut oil submitted to the storage conditions here applied and that these conditions will influence their formation. However, only the first two kinds of compounds have been clearly detected in the samples here studied.

**Figure 10:**
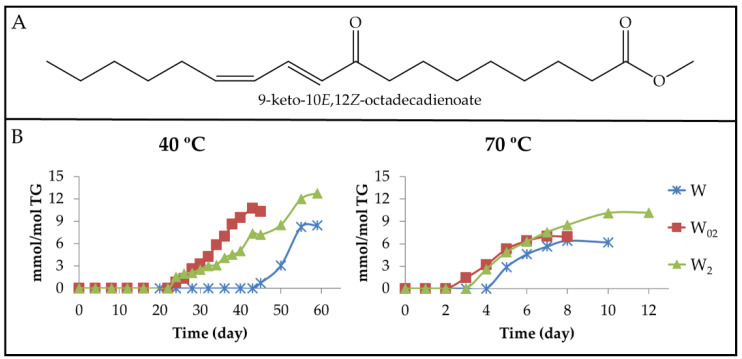
(**A**) Structure of 9-keto-10*E*,12*Z*-octadecadienoate as an example of mKO-c(*Z*,*E*)-dE. (**B**) Evolution of the concentration expressed as mmol/mol TG, of mKO-c(*Z*,*E*)-dEs in walnut oil, with different enrichment levels of αT (W, W_02_ and W_2_), submitted to either 40 °C or 70 °C, up to advanced stages of the oxidation process.

**Figure 11:**
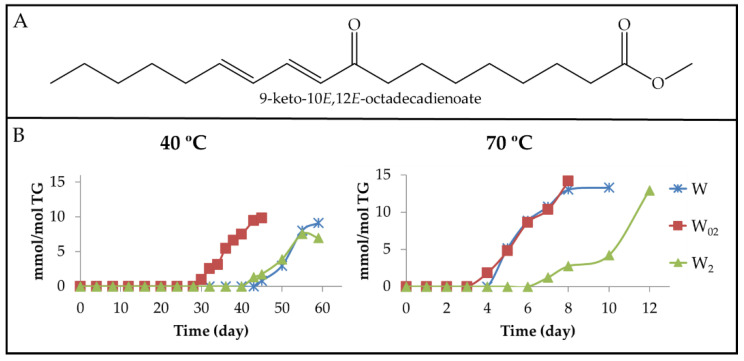
(**A**) Structure of 9-keto-10*E*,12*E*-octadecadienoate as an example of mKO-c(*E*,*E*)-dE. (**B**) Evolution of the concentration expressed as mmol/mol TG, of mKO-c(*E*,*E*)-dEs in walnut oil with different enrichment levels of αT (W, W_02_ and W_2_), submitted to either 40 °C or 70 °C, up to advanced stages of the oxidation process.

(a) Monoketo conjugated dienes (mKO-c-dEs). These derivatives are well-known oxidation compounds. Figure 10A and Figure 11A show the structure of two of them. It has been described that they come from mHPO-c-dEs, keeping either the *Z*,*E* or the *E*,*E* configuration of their corresponding precursor [26]. However, the formation of both kinds of isomers has also been described as coming from only mHPO-c(*Z*,*E*)-dEs [19]. However, these two studies were carried out using pure precursor compounds submitted to very different conditions to those employed here.

Both kinds of mKO-c-dEs have protons giving specific ^1^H NMR non-overlapped signals, as can be seen in Table S8, which are present in the spectra of the different samples in this study after a certain storage time. From the intensity of these specific signals, the concentration of both kinds of derivative, mKO-c(*Z*,*E*)-dEs and mKO-c(*E*,*E*)-dEs, was estimated in each sample throughout the storage time at both temperatures, and the results are represented in Figure 10B and Figure 11B.

From Figure 8B and Figure 10B, it can be observed that although both mKO-c(*Z*,*E*)-dEs and mHO-c(*Z*,*E*)-dEs come presumably from the same precursors (mHPO-c-(*Z*,*E*)-dEs), the onset of formation of the former is delayed in relation to that of the latter, at both temperatures and at any enrichment in αT level. Furthermore, the concentration reached by mKO-c(*Z*,*E*)-dEs in the most αT enriched sample (W_2_), at both temperatures, is between that of the other two samples (W_02_ and W), except at the most advanced oxidation stage, where this sample (W_2_) reaches the highest concentration. Nevertheless, the maximum concentration reached in all cases can be considered low.

As mentioned above, mKO-c(*E*,*E*)-dEs are also formed. Figure 11A shows the structure of one of these compounds and Figure 11B the evolution of their concentrations in all samples at both temperatures. It is noteworthy that the formation onset of these compounds in sample W, as Figure 10B and Figure 11B show, occurs simultaneously to that of their isomers mKO-c(*Z*,*E*)-dEs at both temperatures, as it occurs with the onset of formation of both kinds of mHPO-c-dEs in the same sample (see Figure 3B and Figure 4B).

However, in the W_02_ and W_2_ samples, the formation of mKO-c(*E*,*E*)-dEs is retarded in relation to that of mKO-c(*Z*,*E*)-dEs. This delay is greater the higher the αT level is, as was also observed in the case of both kinds of mHPO-c-dEs (see Figure 3B and Figure 4B). This occurs at both temperatures, but it is much more noticeable at 40 °C than at 70 °C. This delay is associated with the ability of αT to inhibit the isomerization from *Z*,*E* to *E*,*E* as previously mentioned. Finally, it only remains to add that the higher concentration reached by mKO-c(*E*,*E*)-dEs in the samples submitted to 70 °C than in those submitted at 40 °C can be explained by the fact that the increase in temperature favours the isomerization from *Z*,*E* to *E*,*E*, which is required for the formation to these compounds.

**Figure 12:**
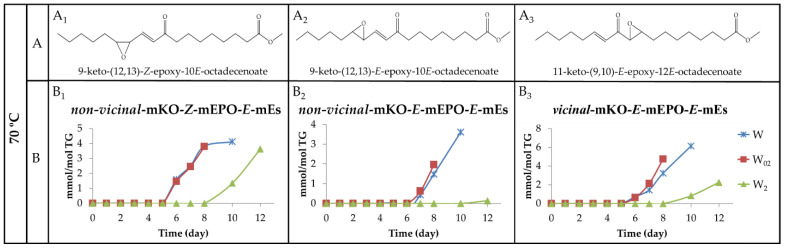
(**A**) Structure of: (**A_1_**) 9-keto-(12,13)-*Z*-epoxy-10*E*-octadecenoate as an example of non-vicinal mKO-*Z*-mEPO-*E*-mE; (**A_2_**) 9-keto-(12,13)-*E*-epoxy-10*E*-octadecenoate as an example of non-vicinal mKO-*E*-mEPO-*E*-mE; (**A_3_**) 11-keto-(9,10)-*E*-epoxy-12*E*-octadecenoate as an example of vicinal mKO-*E*-mEPO-*E*-mEs. (**B**) Evolution of the concentration expressed as mmol/mol TG, of: (**B_1_**) non-vicinal mKO-*Z*-mEPO-*E*-mEs, (**B_2_**) of non-vicinal mKO-*E*-mEPO-*E*-mEs, and (**B_3_**) and of vicinal mKO-*E*-EPO-*E*-mEs, in walnut oil samples having different enrichment level in αT (W, W_02_ and W_2_), submitted to 70 °C up to advanced stages of the oxidation process.

(b) Monoketo monoepoxy monoenes (mKO-mEPO-mEs). Both, mKO-*E*-mEPO-*E*-mEs and mKO-*Z*-mEPO-*E*-mEs, with their oxygenated groups in position either vicinal or non-vicinal, have been described as coming from mHPO-c(*Z*,*E*)-dEs [19]. More recently, their synthesis and ^1^H NMR characterization have also been carried out [68]. Moreover, some of them have been found among the oxidation compounds of corn oil submitted to accelerated storage at 70 °C [30]. Therefore, it is expected that they can also be formed in the walnut oil oxidation subject to study here.

(b_1_) Non-vicinal monoketo monoepoxy *E*-monoenes. Those described are 9-KO-(12,13)-*E*-EPO-10*E*-mE (see Figure 12A_2_ for the structure) and 13-KO-(9,10)-*E*-EPO-11*E*-mE and their corresponding isomers 13-KO-(9,10)-*Z*-EPO-11*E*-mE and 9-KO-(12,13)-*Z*-EPO-10*E*-mE (see in Figure 12A_1_ the structure of this latter compound). All of them are derived from mHPO-c(*Z*,*E*)-dEs. The ^1^H NMR proton signals of these derivatives are given in Table S9, according to Gardner el al., [19], Lin et al., [68], Hidalgo et al., [69] and Ramsden et al. [70]. It can be observed that the spectra of both mKO-*E*-mEPO-*E*-mEs and mKO-*Z*-mEPO-*E*-mEs have a common signal at 3.20 ppm, whereas mKO-*Z*-mEPO-*E*-mEs have two other specific signals at 3.53 ppm and 6.66 ppm, which are absent in the spectrum of mKO-*E*-mEPO-*E*-mEs.

Non-vicinal monoketo *Z*-monoepoxy *E*-monoenes (non-vicinal mKO-*Z*-mEPO-*E*-mEs). The study of the spectra of the different walnut oil samples subjected to 40 °C showed that although the non-vicinal mKO-*Z*-mEPO-*E*-mEs signals centred at 6.66 ppm and 3.53 ppm are distinguishable, after 24 and 26 days of storage onwards in samples W_02_ and W_2_, respectively, and after 43 days onwards in sample W, they have very weak intensity. For this reason, the concentration of these derivatives was not estimated due to the inaccuracy that their estimation involves.

However, as the intensity of the above-mentioned signals is somewhat higher in the spectra of the same samples stored at 70 °C, the estimation of the concentration of these compounds could be carried out and is plotted against the storage time in Figure 12B_1_. It can be observed that the formation onset of these derivatives occurs at the same time in W and W_02_ samples and somewhat later in the W_2_ sample. The evolution paths of non-vicinal mKO-*Z*-mEPO-*E*-mEs are, to a certain extent, similar to those of mKO-c(*E*,*E*)-dEs (see Figure 11B and Figure 12B_1_), with a small delay in the formation onset and with a lower formation rate, which is why the concentration reached by non-vicinal mKO-*Z*-mEPO-*E*-mEs is very low in all the samples, as Figure 12B_1_ shows.

Non-vicinal monoketo *E*-monoepoxy *E*-monoenes (non-vicinal mKO-*E*-mEPO-*E*-mEs). These compounds were not formed in samples stored at 40 °C, as could be inferred from their ^1^H NMR spectra. Nevertheless, they are formed in all the samples submitted to storage at 70 °C, evidencing that the increase in temperature favours their formation. Figure 12A_2_ shows the structure of one of these compounds, whereas in Figure 12B_2_, the evolution of the joint concentration of all these compounds versus storage time is represented. It can be observed that although the concentration is very low in all cases, the highest concentration was reached in the W sample, and the lowest in the W_2_ sample, as could be expected.

(b_2_) Vicinal monoketo monoepoxy *E*-monoenes (vicinal mKO-mEPO-*E*-mEs). These derivatives have only been found in concentrations high enough to be detected by ^1^H NMR in samples submitted to 70 °C. The spectral signals of these compounds are given in Table S9. The intensity of the double triplet signal centred near 7.02 ppm was used for the estimation of their concentration throughout the storage time. Figure 12A_3_ shows the structure of one of these compounds, whereas in Figure 12B_3_, the evolution of the concentration of all these compounds jointly versus storage time is depicted. It can be observed that the evolutions of their paths are, to a certain extent, similar to those of non-vicinal mKO-*E*-mEPO-*E*-mEs (Figure 12B_2_), although their formation rate is somewhat higher.

In summary, and referring to all mKO-mEPO-*E*-mEs, it has been found that the lower the storage temperature, the more their formation is hindered, and this is also true the higher the enrichment level in αT. Both factors are associated with the inhibition of the isomerization from *Z*,*E* to *E*,*E*, as has been repeatedly noted throughout this discussion. Finally, it only remains to add that the concentration reached by these kinds of compounds is very low compared to that of their precursors mHPO-c(*Z*,*E*)-dEs.

#### 3.2.5. Aldehyde Groups, Furan Rings, and Acid Groups Joined Either to Small Molecules or to Truncated Acyl Groups

Simultaneously with the formation of the previously mentioned long chains having several perfectly structured functional groups and responding to specific patterns derived from mHPO-c(*Z*,*E*)-dEs, some other functional groups joined to small molecules, and possibly also to truncated acyl groups, are also formed deriving from some of the previously mentioned long chains. Compounds of small size have been detected as volatile oil components in studies on the evolution of the headspace of edible oils rich in polyunsaturated acyl groups submitted to 70 °C, as is the case here [71]. Among these, there are acids, alcohols, ketones, alkyl furan derivatives, furanones, and aldehydes, some of them having two oxygenated functional groups, and it could be expected that both the αT enrichment degree of walnut oil and storage temperature can influence their formation. Nevertheless, only those compounds detected by ^1^H NMR will be discussed below.

**Figure 13:**
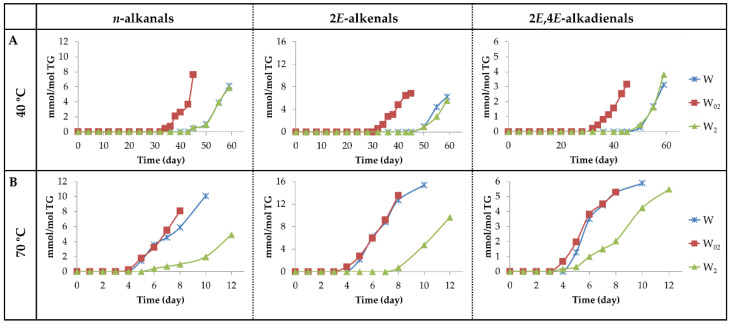
(**A**) Evolution of the concentration expressed as mmol/mol TG, of *n*-alkanals, 2*E*-alkenals, and 2*E*,4*E*-alkadienals in walnut oil with different enrichment levels of αT (W, W_02_ and W_2_) submitted to 40 °C up to advanced stages of the oxidation process; (**B**) the same at 70 °C.

(a) Aldehydes. Several routes have been proposed for the formation of the different types of aldehydes, mHPO-c(*Z*,*E*)-dEs being considered as their precursors that can evolve through different pathways. In some of these pathways, intermediate derivatives such as dHPO-nc(*E*,*E*)-dEs, dHPO-c(*Z*,*E*)-dEs [27,62,72], mHPO-mEPO-*E*-mEs [65] and mHO-c(*Z*,*E*)-dEs [66] have been postulated. It is usually described that two different aldehydes are formed from each long-chain precursor. The identification and quantification of several kinds of aldehydes can be made from the ^1^H NMR spectral signals of their aldehydic protons (see Table S10), as in previous studies [15]. The aldehydes identified include alkanals, aldehydes in unsaturated chains with *E*-double bonds, oxygenated alpha,beta-*E*-unsaturated aldehydes, and *Z*-unsaturated aldehydes. The evolution of the concentration of all of them throughout the storage time in all samples at both temperatures will be discussed below.

(a_1_) Alkanals, 2*E*-alkenals and 2*E*,4*E*-alkadienals. Figure 13 depicts the evolution of the concentration of these aldehydes. It can be observed that upon storage at 40 °C (see Figure 13A), the generation of these aldehydes occurs much earlier in sample W_02_ than in samples W and W_2_.

Regarding the maximum concentrations reached, there are no great differences among these aldehydes in the different samples, even though 2*E*,4*E*-alkadienals reach lower concentrations than the other two. From the observation of Figure 3B, Figure 11B and Figure 13A, it seems evident that the factors governing the formation of these derivatives at 40 °C are the same as those mentioned in the formation of mHPO-c(*E*,*E*)-dEs and of mKO-c(*E*,*E*)-dEs. Once the parent precursors, mHPO-c(*Z*,*E*)-dEs, are formed, their stability and the inhibitory capacity of the system for the isomerization from *Z*,*E* to *E*,*E* appear to be determining factors in the formation of these aldehydes, both being dependent on the enrichment level in αT.

These same factors also influence the formation of these aldehydes at 70 °C (see Figure 4B, Figure 11B and Figure 13B). In addition, their formation is also affected by the temperature increase from 40 °C to 70 °C. This temperature increase accelerates the formation of the precursors, especially in the W sample, reduces their stability and favours the isomerization from *Z*,*E* to *E*,*E*, the latter two effects being contrary to those caused by αT enrichment.

**Figure 14:**
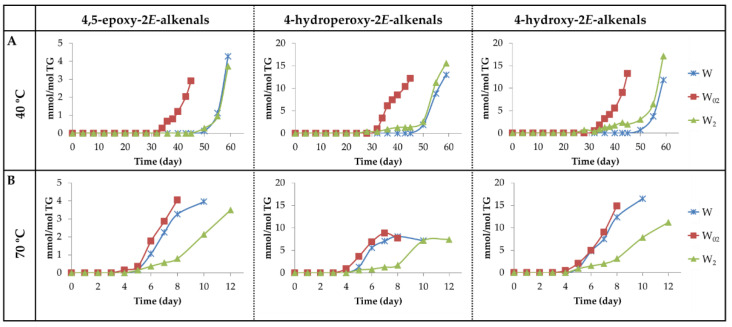
(**A**) Evolution of the concentration expressed as mmol/mol TG, of 4,5-EPO-2*E*-alkenals, 4-HPO-2*E*-alkenals and 4-HO-2*E*-alkenals in walnut oil with different enrichment levels of αT (W, W_02_ and W_2_) submitted to 40 °C up to advanced stages of the oxidation process. (**B**) The same at 70 °C.

As a consequence, the formation onset of these three types of aldehydes at 70 °C occurs at almost the same storage time in samples W_02_ and W and somewhat later in sample W_2_, similarly to what occurred in the formation at this temperature of mHPO-c(*E*,*E*)-dEs, non-vicinal mHPO-mEPO-mEs, mKO-c(*E*,*E*)-dEs and mKO-mEPO-*E*-mEs. Moreover, the rates of formation of these aldehydes are of a similar order in samples W and W_02_ and somewhat lower in W_2_, showing that, although the increase in temperature favours isomerization from *Z*,*E* to *E*,*E*, the inhibitory effect of αT against this isomerization has a clear influence in the most enriched sample. Furthermore, it is noticeable that at this temperature, the three kinds of aldehydes are formed in higher concentration than at 40 °C, this effect being more pronounced in 2*E*-alkenals and in the W sample.

(a_2_) Oxygenated alpha,beta-*E*-unsaturated aldehydes. This group includes 4,5-EPO-2*E*-alkenals, 4-HPO-2*E*-alkenals and 4-HO-2*E*-alkenals. As shown in Figure 13A,B and Figure 14A,B, the evolution of the concentration of 4,5-EPO-2*E*-alkenals, at both temperatures in all the samples, resembles that of the non-oxygenated aldehydes mentioned above, especially that of the 2*E*,4*E*-alkadienals. It should be remembered that these latter aldehydes have been described as the main intermediate compounds in the formation of 4,5-EPO-2*E*-alkenals by epoxidation [65,73].

The onset of formation of the other two types of aldehydes, 4-HPO- and 4-HO-2*E*-alkenals, at 40 °C occurs almost at the same time in samples W_2_ and W_02_, and later in the W sample. However, their concentration in the W_2_ sample remains very low for an extended period of time, showing an evolution similar to that dHPO-nc(*E*,*E*)-dEs and non-vicinal mHPO-mEPO-*E*-mEs (see Figure 6B, Figure 7B and Figure 14A), which increases significantly in the later stages of the oxidation process. The similarity might suggest that either the latter are intermediate precursors of the former, or the formation of all of them is influenced in the same direction by the same factors. It should be mentioned that dHPO-nc(*E*,*E*)-dEs have been proposed, in previous studies, as intermediate derivatives in the formation of these aldehydes [27,62,72]. The fact that the formation of these aldehydes occurs earlier in the sample most enriched in αT is of great relevance due to the toxicity attributed to them [5,74]. Nevertheless, it has been described that some of them, such as 4-HO-2*E*-nonenal at low concentration, are not considered toxic, but they display several physiological activities [75,76,77,78,79,80,81].

**Figure 15:**
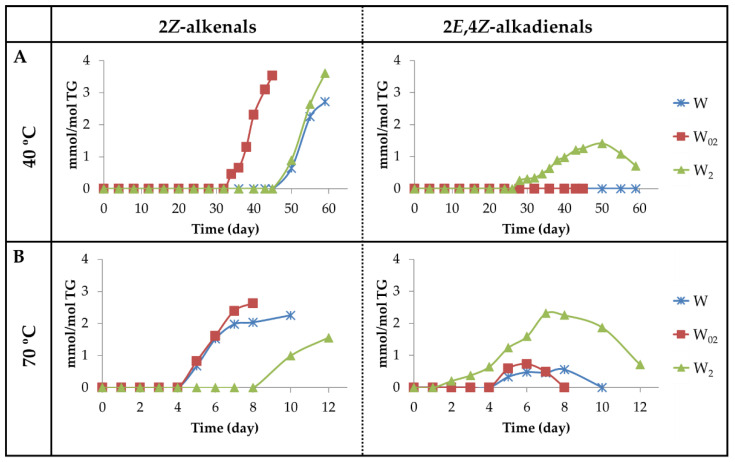
(**A**) Evolution of the concentration expressed as mmol/mol TG, of 2*Z*-alkenals giving ^1^H NMR signals near 10.05–10.07 ppm and 2*E*,4*Z*-alkadienals in walnut oil with different enrichment levels of αT (W, W_02_ and W_2_) submitted to 40 °C up to advanced stages of the oxidation process. (**B**) The same at 70 °C.

In the storage at 70 °C, the onset of the formation of these three kinds of aldehydes is almost coincident. However, their formation rate in sample W_2_ is much slower than in the other two, as occurs in the formation of dHPO-nc(*E*,*E*)-dEs and non-vicinal mHPO-mEPO-*E*-mEs at this temperature.

(a_3_) Aldehydes having *Z*-unsaturated bonds. Two kinds of aldehydes have been included in this group. One of them is constituted by 2*Z*-alkenals giving a doublet signal around 10.05–10.07 ppm (see Table S10) [82]. In previous studies, 2*Z*-alkenals have been found as small molecules, among the volatile oxidation compounds formed from 9-HPO-10*E*,12*Z*-dEs or 13-HPO-9*Z*,11*E*-dEs submitted to different conditions, so their formation in the samples studied here is to be expected [73,83,84]. The evolution of the concentration of these aldehydes over the storage time, at both temperatures, has been estimated from the intensity of the signal mentioned above and is depicted in Figure 15A,B. The fact that the onset of formation of 2*Z*-alkenals is similar to that of 2*E*-alkenals in all samples at both temperatures, as Figure 13A,B and Figure 15A,B show, suggests that their formation could be related. In fact, as Figure 13A,B show, the maximum concentration reached by 2*E*-alkenals is approximately double at 70 °C than at 40 °C, whereas the opposite is true for 2*Z*-alkenals, as Figure 15A,B show. This agrees with the higher thermodynamic stability of the first in relation to the second ones and with the higher kinetic stability of the seconds with respect to the first ones.

The other kind of aldehydes included in this group are 2*E*,4*Z*-alkadienals, which possibly come directly from mHPO-c(*Z*,*E*)-dEs, as 2*Z*-alkenals. Those that are not attached to long-chain molecules have been detected among the volatile components of edible oils rich in polyunsaturated groups submitted to oxidation [71]. The concentration of these aldehydes over the storage time was estimated from the intensity of their aldehydic proton signal (see Table S10) and has been depicted versus the storage time in Figure 15A,B. It is worth noting that in the storage at 40 °C, these aldehydes are only detected by ^1^H NMR in the sample most enriched in αT (W_2_). However, at 70 °C, they are detected in the three samples, W, W_02_ and W_2_, although in the first two samples at very low concentrations. It is also worth noting that, as mentioned previously, 2*E*,4*Z*-alkadienals together with 4-HPO- and 4-HO-2*E*-alkenals are the first aldehydes formed in the sample most enriched in αT, W_2_, at both temperatures.

**Figure 16:**
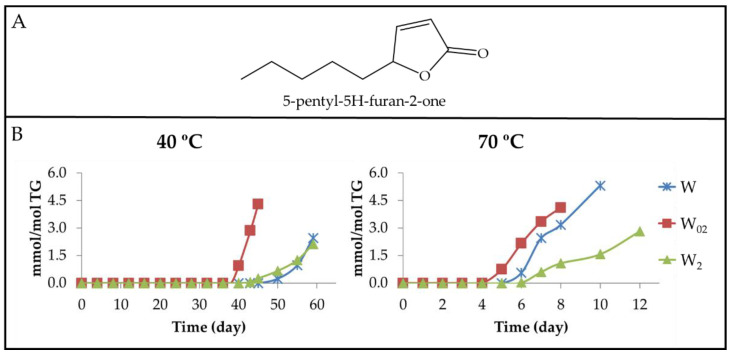
(**A**) Structure of one 5-alkyl-5H-furan-2-one. (**B**) Evolution of the concentration expressed as mmol/mol TG, of 5-alkyl-5H-furan-2-ones in walnut oil with different enrichment levels of αT (W, W_02_ and W_2_), submitted to either 40 °C or 70 °C up to advanced stages of the oxidation process.

(b) Furan derivatives. As mentioned above, alkyl-furans and 5-alkyl-2(5*H*)-furanones have been detected in the headspace of edible oils submitted to oxidative conditions [71,85,86]. However, only when their concentration in the sample is enough to be detected by ^1^H NMR can they be identified and quantified by this technique.

(b_1_) Alkyl furans. Within this group of compounds, the main are 5-alkyl furans, such as 5-pentyl furan [71], whose protons give a characteristic non-overlapped ^1^H NMR signal (see Table S11). They have been described to be formed in the oxidation of mHPO-c(*Z*,*E*)-dEs [73] or of mHO-c(*Z*,*E*)-dEs [69]. However, in the samples studied here, due to their characteristic signal only is detected at the end of the storage in all cases and with very low intensity, they were not quantified. However, it is worth noting that these compounds are very well known among the edible oil volatile oxidation compounds [71].

(b_2_) Alkyl furanones. These are also compounds well known among the volatile derivatives coming from edible oil oxidation, the main ones being 5-alkyl-2(5H)-furanones (see Figure 16A) [85,86]. The evolution of their concentration throughout the storage time has been estimated in all samples at both temperatures from the intensity of the ^1^H NMR signal centred at 7.46 ppm, as shown in Table S11. The results obtained are depicted in Figure 16B.

It can be observed in Figure 14A,B and Figure 16B that the evolution of their concentration is, to a certain extent, similar to that of several aldehydes, although in some cases, the onset of the formation of 5-alkyl-2(5H)-furanones occurs later.

(c) Short-chain acids. Formic acid. As already mentioned, in addition to aldehydes and furan derivatives, among the components of the headspace of edible oils subjected to oxidative conditions [71,86,87], acids, alcohols and even esters of small size have also been found. Their formation can occur by different pathways [62,88].

Among the various acids of small size that can be formed, one that stands out by its concentration at advanced stages of the oxidation process is formic acid [71,86,88,89]. Although the ^1^H NMR singlet signal of its carbinol methine proton was detected long ago [90], it was not until recently that it was correctly assigned [30]. Surprisingly, this ^1^H NMR singlet signal does not appear in any of the spectra of the walnut oil samples studied here. However, this cannot be attributed to formic acid not being formed during oxidation of this oil, but to other reasons. The absence of this signal in oxidized walnut oil spectra could be due to possible interactions between formic acid and pyrazine, pyridine and some of their derivatives, which are present in this walnut oil due to the roasting of the nuts prior to oil extraction [87], which could prevent the appearance of this signal. In fact, studies carried out in our laboratory by means of ^1^H NMR about the oxidation of nut oils in which their previous processing included roasting or not have shown that the singlet signal of formic acid only appears in the oxidized oils coming from walnuts that were not roasted. Furthermore, the fact that formic acid derivatives are present in the different oxidized walnut oil samples studied here, as will be explained below, is evidence of the presence of formic acid in the several oxidized walnut oil samples.

The importance of the formation of acids and alcohols in outstanding concentration at advanced stages of the oxidation process lies in the fact that they may react with other oil components, thereby influencing the course of the oxidation process.

Finally, it only remains to add that the formation pathways of the structures described in Section 3.2.1, Section 3.2.2, Section 3.2.3, Section 3.2.4 and Section 3.2.5, supposedly derived from mHPO-c(*Z*,*E*)-dEs, could be similar to those occurring in the enzymatic oxidation provoked by the action of lipoxygenases on the same substrate [91]. However, to the best of our knowledge, in enzymatic oxidation processes, which can also occur endogenously, no such high number of oxylipins has been identified until now. Furthermore, it is worth noting that some of the oxylipins mentioned above, such as dihydroperoxy non-conjugated dienes, non-vicinal monohydroperoxy monoepoxy monoenes, non-vicinal monohydro monoepoxy monoenes and vicinal and non-vicinal monoketo monoepoxy monoenes, which were recently detected for the first time in the oxidation process of vegetable oils [30], have also been formed in the oxidation process undergone by the W, W_02_ and W_2_ samples during storage at both temperatures. Furthermore, this study not only reinforces and gives soundness to the previous results, but it has also addressed, for the first time, both separately and jointly, the effect of the storage temperature and αT enrichment degree in the formation of these oxylipins in an oil rich in polyunsaturated acyl groups.

#### 3.2.6. Other Oxygenated Functional Groups Attached to Long Chains Formed in Advanced Stages of the Oxidation Process

Figure 2 and Figure 5 show that when the concentration of hydroperoxy groups decreases, the degradation rate of polyunsaturated acyl groups remains high, which could suggest that at this stage of the oxidation process, there are other routes of degradation of acyl groups different to those based on the formation of mHPO-c(*Z*,*E*)-dEs mentioned previously. Furthermore, when hydroperoxy groups are present in a significant concentration, the oil also contains a certain concentration of acids, such as formic, acetic, and hexanoic, as well as of alcohols, among other compounds [71] which can favour certain reactions in the polyunsaturated acyl groups, generating epoxy, vicinal dihydroxy, ester or ether groups.

**Figure 17:**
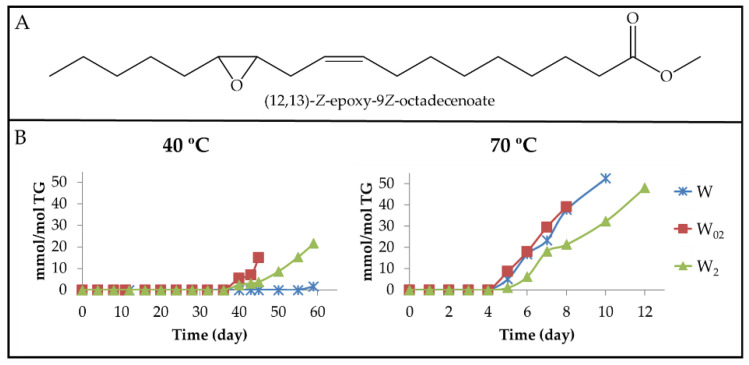
(**A**) Structure of (12,13)-*Z*-epoxy-9*Z*-octadecenoate as an example of *Z*-mEPO-*Z*-mE. (**B**) Evolution of the concentration expressed as mmol/mol TG, of *Z*-mEPO-*Z*-mEs plus *Z*-mEPO-*Z*-dEs present in walnut oil having different enrichment levels in αT (W, W_02_ and W_2_), submitted to either 40 °C or 70 °C up to advanced stages of the oxidation process.

(a) Long-chain derivatives supporting epoxy groups (EPOs). The possibility of epoxidation reactions occurring directly on the unmodified acyl groups could be envisaged because there are conditions for this in advanced stages of the storage process. These conditions are the presence of strong oxidants, in this case hydroperoxides, and the presence of acids such as formic acid, among others [92,93,94]. In addition, it should be noted that even after a long period of storage under oxidative conditions, a certain percentage of unmodified polyunsaturated acyl groups remains in the walnut oil samples. Among the long-chain derivatives having epoxy groups that can be formed, are *Z*-EPOs and *E*-EPOs.

(a_1_) Long-chain derivatives supporting *Z*-epoxy groups (*Z*-mEPOs). Although in the oxidation process of walnut oil, the formation of *Z*-mono, *Z*-di, or even *Z*-triEPOs groups in long chains could be expected due to its content in linoleic and linolenic groups, these possibilities are reduced to only *Z*-mEPOs. This is proved by the absence of the ^1^H NMR signals, from 1.70 ppm to 1.85 ppm, due to methylene protons in the alpha position regarding two EPO groups (see Tables S12 and S13), and near 3.0 ppm, of the external oxirane ring protons of *Z*-di or *Z*-triEPOs. This shows that only long-chain derivatives with *Z*-mEPOs groups are formed in enough concentration to be detected by ^1^H NMR spectroscopy [29,93]. Among these compounds, there are the well-known (9,10)-*Z*-EPO-12*Z*-octadecenoate and (12,13)-*Z*-EPO-9*Z*-octadecenoate, also named leukotoxin and isoleukotoxin, respectively, the latter being shown in Figure 17A.

The estimation of the concentration of *Z*-mEPOs long-chain derivatives is not straightforward because several compounds give signals that overlap with that of these epoxy protons (see Tables S3, S9 and S10) and these contributions have to be subtracted. The signals that overlap with that of the epoxydic protons of *Z*-mEPOs long-chain derivatives belong to some mKO-mEPO-mEs (see Table S9), to 4,5-EPO-2*E*-alkenals (see Table S10), and to mHPO-c(*Z*,*E*)-tEs (see Table S3). Furthermore, the side bands of the bis-allylic methylene protons of linolenic and linoleic acyl groups also overlap with this signal. Taking all these considerations into account, the approached estimation of the concentration, throughout the storage time, mainly of *Z*-mEPO-*Z*-mEs, was carried out and the results are depicted in Figure 17B. It can be observed that the formation of these derivatives is more favoured at 70 °C than at 40 °C.

(a_2_) Long-chain derivatives supporting *E*-epoxy groups (*E*-mEPOs). In addition to the formation of *Z*-mEPOs long-chain derivatives, the formation of the *E*-mEPOs counterparts is also possible. Thus, the spectra of some walnut oil samples show a multiplet signal centred near 2.70 ppm attributable to the *E*-EPOs protons of (9,10)-*E*-EPO-12*Z*-octadecenoate, in agreement with that of the corresponding standard compound and with data provided by Nilewski et al. [95] for the same compounds.

**Figure 18:**
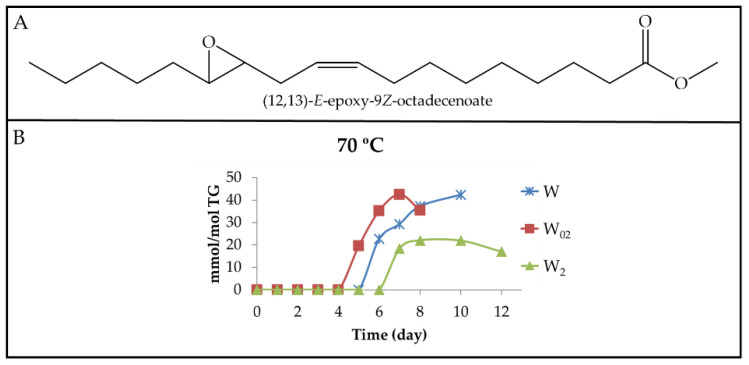
(**A**) Structure of (12,13)-*E*-epoxy-9*Z*-octadecenoate as an example of *E*-mEPO-*Z*-mE. (**B**) Evolution of the concentration expressed as mmol/mol TG, of *E*-mEPO-*Z*-mEs in walnut oil with different enrichment levels of αT (W, W_02_ and W_2_), submitted to 70 °C up to advanced stages of the oxidation process.

Figure 18A gives the structure of one of these compounds, and Figure 18B shows the evolution of the concentration of this kind of derivative in the samples submitted to 70 °C. This signal has not been found in the spectra of any of the samples stored at 40 °C, which indicates the important role of the temperature in its formation.

Finally, it only remains to be added that it has been reported that mEPOs long-chain derivatives can yield, after oxirane ring opening and chain breaking, two aldehyde molecules, contributing to the formation of these derivatives, and which have been described extensively in Section 3.2.4 [96,97,98]. Furthermore, it has also been described that mEPOs long-chain derivatives after oxirane ring opening are the starting point for the formation of other long-chain derivatives, some of which are responsible for oil polymerization and will be addressed below [93,99].

**Figure 19:**
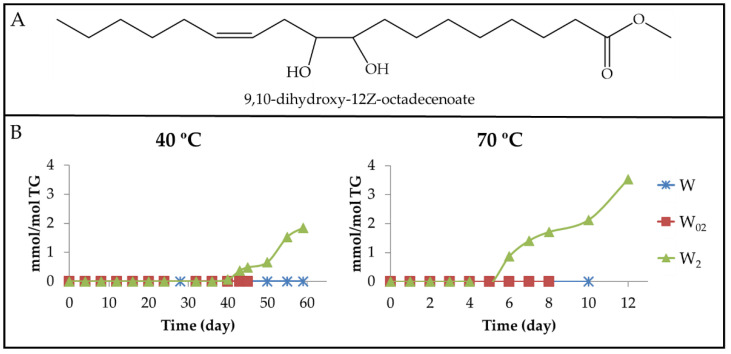
(**A**) Structure of 9,10-dihydroxy-12*Z*-octadecenoate as an example of a long-chain derivative supporting a vicinal dHO group. (**B**) Evolution of the concentration expressed as mmol/mol TG, of vicinal dHO supported in long chains in walnut oil with different enrichment levels of αT (W, W_02_ and W_2_) submitted to either 40 °C or 70 °C, up to advanced stages of the oxidation process.

(b) Long-chain derivatives supporting oxygenated functional groups formed in the oxirane ring opening. The opening of the oxirane ring by simple hydrolysis or by reaction with acids or with alcohols yields new functional groups.

(b_1_) Long-chain derivatives supporting vicinal dihydroxy groups (vicinal dHO). The opening of the oxirane ring caused by hydrolysis produces two vicinal hydroxy groups for each hydrolyzed oxirane ring [100]. This reaction leads to the formation of polyols in highly epoxidised oils, and it is the starting point for the formation of polymers of industrial interest from vegetable origin [101,102]. Taking into account that, in the samples under study here, only mEPOs in long chains have been found, only the formation of long-chain derivatives with two vicinal hydroxy groups could be expected. In Figure 19A, an example of a vicinal dHO long-chain derivative is represented. The well-known toxic diols coming from either leukotoxin or isoleukotoxin mentioned previously belong to this group.

These derivatives, in addition to other ^1^H NMR signals common to many other compounds, have a characteristic signal due to the secondary alcohol carbinol methine proton. This signal, which is broad in the spectrum of methyl 12,13-dHO-9*Z*-octadenoate, is centred around 3.42 ppm, while in that of methyl 9,10-dHO-12*Z*-octadecenoate, it is centred around 3.45 ppm [103]. Very similar chemical shifts and multiplicities to the above have been reported in other studies for the signals of the same type of protons of the corresponding fatty acids [95].

In the spectra of the samples studied here, a broad signal centred near 3.43 ppm that could be attributed to these derivatives appears. However, epoxidic protons of non-vicinal mHPO- and of non-vicinal mHO-*Z*-mEPO-*E*-mEs also contribute to this signal, as Tables S5 and S7 show. Taking into account these contributions, an approximate estimation of the concentration of long-chain derivatives supporting vicinal dOH groups along the storage time was made and is depicted in Figure 19B.

**Figure 20:**
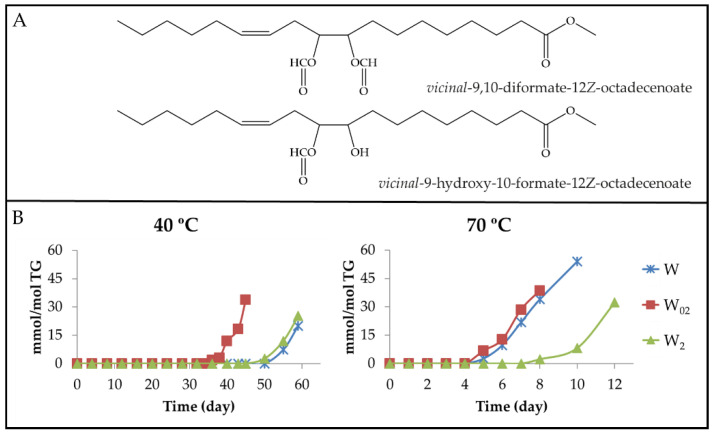
(**A**) Structure of an example of long-chain derivatives supporting vicinal dF and vicinal mF-mOH groups. (**B**) Evolution of the concentration expressed as mmol/mol TG, of total formate groups in walnut oil samples having different enrichment level in αT (W, W_02_ and W_2_), submitted to either 40 °C or 70 °C up to advanced stages of the oxidation process.

It can be observed that these vicinal dOH derivatives are only formed, in enough concentration to be detected by ^1^H NMR, in the walnut oil samples most enriched in αT at both temperatures, the concentration reached at 70 °C being higher than at 40 °C, in agreement with that happening in the formation of their precursors *Z*-mEPOs. Finally, it only remains to recall that vicinal dOH derivatives have been postulated as intermediate compounds in the formation of aldehydes from epoxides under certain conditions [98].

(b_2_) Long-chain derivatives supporting either two vicinal ester groups (vicinal dEst) or vicinal monoester monohydroxy groups (vicinal mEst-mHO). Formates (F). The oxirane ring opening mediated by acids can result in the formation of structures such as those shown in Figure 20A, either with two vicinal dEst groups or with vicinal mEst-mHO groups per disappeared epoxy group. This reaction is well known and has been widely used as a first step to obtain polymers from highly epoxidized vegetable oils, giving rise to different types of polyesters or polyformates. The formation of one or the other depends on the nature of the unsaturated acyl groups of the starting oil and on the acids used in the process, the use of formic acid being very common [104,105,106,107].

The occurrence of this type of reaction in the oxidation process undergone by the samples studied here is proved by the presence in their spectra of both the typical signals between 8.00 ppm and 8.17 ppm due to the carbinol methine protons of the formate group, and the signals between 3.80 ppm and 4.04 ppm (at 3.85 ppm, 3.93 ppm, 3.98 ppm and 4.04 ppm) attributed to the carbinol methine protons of the secondary alcohol group vicinal to the formate or ester group [104,106]. Signals from this second region appear in the spectra of the walnut samples simultaneously with those of the first region. From the intensity of the signals between 8.05 ppm and 8.17 ppm, the concentration of total formate groups throughout the storage process in the different samples has been estimated and is represented in Figure 20B.

The formation onset of these derivatives, as shown in Figure 20B, occurs in all cases at advanced stages of the oxidation process, close to that *n*-alkanals and 2*E*-alkenals and with similar paths but with a much higher formation rate. For this reason, they reach much higher concentrations, these always being greater at 70 °C than at 40 °C. It must be remembered that both aldehydes and formates can come from long-chain derivatives supporting epoxy groups [98].

**Figure 21:**
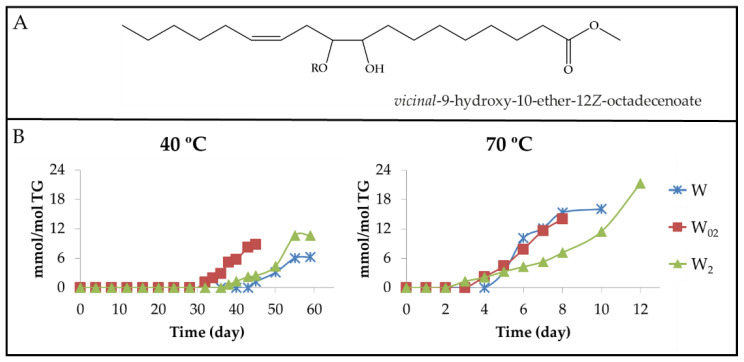
(**A**) Structure of an example of vicinal mEt-mHO derivatives. (**B**) Evolution of the concentration of protons generating the signal at 3.62 ppm attributable to methine protons of ether bonds and of secondary alcohols expressed as mmol/mol TG in walnut oil samples with different enrichment levels in αT (W, W_02_ and W_2_), submitted to either 40 °C or 70 °C up to advanced stages of the oxidation process.

This type of reaction can also take place with other acids present in the oil at advanced stages of the oxidation process, giving rise to ester-type bonds and generating branching in the long chains which contributes to an increase in the oil viscosity.

(b_3_) Long chains supporting either two vicinal ether groups (vicinal dEt) or vicinal monoether monohydroxy groups (vicinal mEt-mHO). The formation of these structures (see Figure 21A) occurs when alcohols provoke the oxirane ring opening. This kind of hydrolysis is possible because it is known that primary alcohols of small size are also present in edible oils submitted to storage as here [71]. Furthermore, as has been shown in this study, different secondary alcohols are present in many of the derivatives detected in all walnut oil samples studied here.

This opening of the oxirane ring can yield, as shown in Figure 21A, a secondary alcohol and the branching of the fatty acyl group by the formation of an ether bond with the alcohol chain. However, when the opening of the oxirane ring is produced by a secondary alcohol attached to a fatty acyl group, this will produce ether linkages between different fatty acyl groups of the same or of different triglycerides, thus contributing to the oil polymerization.

Although the ^1^H NMR signal near 3.62 ppm can be associated with primary alcohols [50], and in vegetable oils submitted to these conditions, small-sized primary alcohols are present, their concentration is much smaller than that of acids [71]. For this reason, and assuming that this signal is mainly due to methine protons of ether bonds of potential oligoether backbones [108,109] and to methine protons of certain secondary alcohols [110], their joint concentration has been determined and represented in Figure 21B in order to have an approximate idea about the evolution of the concentration of these groups. As can be observed, these derivatives are formed in higher concentrations at 70 °C than at 40 °C, as occurs in other derivatives coming from the oxirane ring opening, and the beginning of their formation onset is similar to that of other derivatives coming from the same precursors.

Finally, it should be mentioned that all oxylipins described in Section 3.2.6 could also be formed enzymatically in the oxidation pathway initiated by cytochrome P450 epoxygenase, on either polyunsaturated fatty acids or fatty acyl groups, mEPO-mEs being the parent structure [91,111]. It is known that epoxyde hydrolases are able to open the oxirane ring of mEPO-mEs, yielding the well-known diols. Therefore, it could also occur that the enzymatically formed oxirane ring could be opened by acids or alcohols, giving rise to ester or ether bonds, capable of producing polymerizations between fatty acids or between chains of fatty acyl groups that would confer rigidity to the system involved, as has been observed in this study. The formation of these oxylipins was recently described for the first time in the oxidation of edible oils [30] and their detection in all samples studied here at both temperatures reinforces and gives soundness to the previous results. In addition, the influence of the temperature and of the enrichment degree in αT of the oil in the formation of these oxylipins is shown here for the first time.

### 3.3. Evolution of αT Concentration in the Enriched Walnut Oil Samples throughout the Storage and Effect

As is to be expected, as the walnut oil oxidation advances, the added αT is degraded, and it is also to be expected that this degradation goes parallel to the oil degradation.

**Figure 22:**
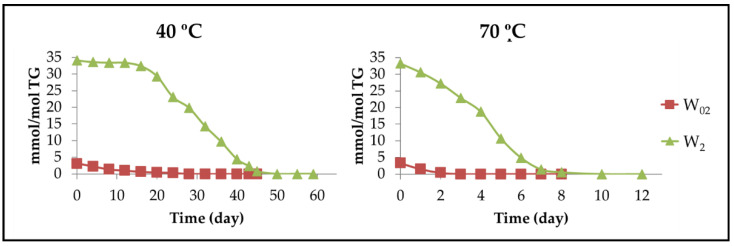
Evolution of the concentration of αT in the several walnut oil samples, initially having two different enrichment levels in αT (W_02_ and W_2_) submitted either to 40 °C or to 70 °C up to advanced stages of the oxidation process.

Thus, in the W_02_ sample, as Figure 2 and Table 1 show, both linoleic and linolenic groups have two clearly differentiated degradation rates throughout storage, the first being smaller than the second one. The time at which the degradation rate of both acyl groups changes coincides with the time at which αT is totally degraded (see Figure 22). This occurs in sample W_02_ near day 26 at 40 °C and near day 3 at 70 °C. The same is true for the degradation rate of linoleic groups in the W_2_ sample. In this case, as Figure 2 and Table 1 show, the changes in the degradation rates of linoleic groups occur from day 45 onwards at 40 °C, and from day 8 onwards at 70 °C, and they coincide with the extinction of αT in the system (see Figure 22). Likewise, in the oil sample W, not enriched in αT, linoleic and linolenic groups have two clearly differentiated degradation rates (see Figure 2 and Table 1), the former being much smaller than the latter. Nevertheless, the degradation rates of the abovementioned acyl groups in W_02_ and W_2_, in the first stage (up to αT total disappearance) is greater the higher the αT enrichment degree and always higher than in the W sample. In contrast, the opposite is true in the second stage, the degradation rate in the W sample being higher than in the other two samples (see Figure 2 and Figure 22 and Table 1).

These results suggest that αT accelerates oil degradation to a greater extent the higher the αT level, showing a clear pro-oxidant behaviour. However, after the disappearance of αT, a delay in the oil degradation occurs to a greater extent the higher the initial concentration of αT in the sample. This suggest that the intermediate compounds formed have more stability the higher the initial concentration of αT in the sample.

### 3.4. Final Considerations Regarding the Factors That Govern the Walnut Oil Oxidation Process and Their Complex Effects

From the results discussed above, it is clear that both the αT concentration and temperature jointly govern the oxidation process of walnut oil with very different effects. Both reduce the oxidative stability of the oil by favouring oxidation but in very different ways. While αT lengthens the duration of the oxidation process, the increase in temperature causes the oxidation process to be shortened. This is commented on below.

In the sample not enriched in αT, W, submitted at 40 °C, in a first stage, the oxidation process advances very slowly for a prolonged period of time, showing a very broad induction period. However, after this induction period, the process evolves very quickly, forming mHPO-c(*Z*,*E*)-dEs and mHPO-c(*E*,*E*)-dEs almost simultaneously. Both kinds of hydroperoxides reach the maximum concentration simultaneously, that of the second being slightly higher than that of the first, both types of primary oxidation compounds showing similar degradation rates. However, the oxidation process during storage at 70 °C is very fast, having a very short induction period. At this temperature, both mHPO-c(*Z*,*E*)-dEs and mHPO-c(*E*,*E*)-dEs also reach the maximum concentration simultaneously, that of the latter being higher than that of the former, both degrading simultaneously. Thus, as is well known, it follows that the higher the temperature, the higher the oxidation rate and the easier the isomerization from *Z*,*E* to *E*,*E*.

In the sample less enriched in αT, W_02_, submitted at 40 °C, the evolution of the oxidation process is very different from that occurring in W. Enrichment in αT causes several effects, among which the following are noteworthy: reduction in the induction period to the minimum expression in relation to that observed in W; formation exclusively of mHPO-c(*Z*,*E*)-dEs during an extended period of time, reaching a significant concentration before the onset of mHPO-c(*E*,*E*)-dEs formation; and maintenance of the concentration of the former higher than that of the latter at all times. The evolution of the oxidation process in W_02_ submitted at 70 °C is also very quick, almost without an induction period. At this temperature, mHPO-c(*Z*,*E*)-dEs are also the only hydroperoxides present during a certain period of time, after which mHPO-c(*E*,*E*)-dEs are formed, these reaching even higher concentrations than their previously mentioned counterparts. The temperature increase provokes the acceleration of the oxidation process of W_02_, as in the case of W, and in addition, an easier isomerization from *Z*,*E* to *E*,*E*.

In the sample most enriched in αT, W_2_, submitted to 40 °C, the induction period is almost absent, generating from the beginning important concentrations of mHPO-c(*Z*,*E*)-dEs which reach very high concentration compared with that reached in W and W_02_ samples. Furthermore, the formation of mHPO-c(*E*,*E*)-dEs is delayed up to the degradation onset of their *Z*,*E* counterparts. The effect provoked on the three aspects previously mentioned by αT enrichment is especially noteworthy in this case. The increase in the temperature in this oil sample also causes a significant acceleration of the oxidation process, and although it favours the isomerization from *Z*,*E* to *E*,*E*, the formation of the latter isomers does not begin until at an advanced stage of the oxidation process.

The effects that a temperature increase and αT enrichment provoke in the formation of primary oxidation compounds are also transferred to the formation of secondary or further oxidation derivatives. Due to this, in some cases, there are similarities in the formation paths of certain derivatives in very different oil samples. Thus, in the process at 40 °C, there is coincidence in the formation onset of certain derivatives in W_02_ and W_2_ samples, in some cases this even being something occurring earlier in W_2_ than in W_02_, and always earlier than in W. These derivatives are mHPO-c(*Z*,*E*)-dEs, mHPO-c(*Z*,*E*)-tEs, mHO-c(*Z*,*E*)-dEs, and mKO-c(*Z*,*E*)-dEs, which reach higher concentrations in W_2_ than in W_02_, although in the case of the last derivative with a much lower formation rate in W_2_ than in W_02_. dHPO-nc(*E*,*E*)-dEs, non-vicinal mHPO-mEPO-mEs, *Z*-mEPO-mEs, 4-HPO-2*E*-alkenals and 4-HO-2*E*-alkenals also belong to this group but reach much smaller concentrations in most cases in W_2_ than in W_02_. In the oxidation process at 40 °C, there is also agreement in the formation onset of other structures in W and W_2_ samples, which is delayed in relation to that found in W_02_. mHPO-c(*E*,*E*)-dEs, non-vicinal mHO-mEPO-mEs, mKO-c(*E*,*E*)-dEs, *n*-alkanals, 2*E*-alkenals, 2*E*,4*E*-alkadienals, 4,5-EPO-2*E*-alkenals, 2*Z*-alkenals, 5-alkyl-5*H*-furan-2-ones, and formats belong to this group. The rate of formation of these structures is in most cases of a similar order in W and W_2_ samples.

In the processes at 70 °C, there is also certain concordance in the formation paths of some derivatives in W_02_ and W_2_ samples. Thus, the formation onset of mHPO-c(*Z*,*E*)-dEs, mHPO-c(*Z*,*E*)-tEs, mHO-c(*Z*,*E*)-dEs, mKO-c(*Z*,*E*)-dEs, and 2*E*,4*Z*-alkadienals is similar in W_2_ and W_02_, although in some cases, earlier in W_2_ than in W_02_, and always earlier than in W. These derivatives, as in the storage at 40 °C, in most cases reach higher concentrations in W_2_ than in W_02_ and W. There is another group of derivatives exhibiting similar formation onset in the three samples but with a much lower formation rate in W_2_. Non-vicinal mHO-mEPO-mEs, *n*-alkanals, 2*E*,4*E*-alkadienals, 4-HPO-2*E*-alkenals, 4-HO-2*E*-alkenals and 4,5-EPO-2*E*-alkenals belong to this group. Finally, there is a third group of derivatives that shows a similar formation onset in W and W_02_, always earlier than in W_2_, with a higher formation rate than in the latter sample. mHPO-c(*E*,*E*)-dEs, dHPO-nc(*E*,*E*)-dEs, non-vicinal mHPO-mEPO-mEs, mKO-c(*E*,*E*)-dEs, mEPO-mEs and formats belong to this group.

In summary, at both temperatures, the αT enrichment accelerates the oxidation of fatty acyl groups by the early formation of oxygenated *Z*,*E*-conjugated dienic structures and related, and delays, regarding the formation of the former, the formation of oxygenated *E*,*E*-conjugated dienic structures and of some related structures. This way, the oxidation process is more extended the greater the degree of enrichment in αT. Moreover, αT enrichment causes an increase in the maximum concentration reached only by oxygenated conjugated *Z*,*E*-diene structures, but not in that of related structures, nor in that reached by oxygenated *E*,*E*-conjugated diene and related structures, which in most cases is even lower. These results prove that αT leads the oxidation process of this unsaturated oil, favouring the formation of some oxylipins and hindering the formation of other ones.

Furthermore, these results also show that the elucidation of the behaviour of certain compounds such as αT, as antioxidant or pro-oxidant, due to the complexity of its effect, requires an in-depth study of the oxidation process. It has been proved that αT enrichment can accelerate the formation of some oxidation compounds, including some primary oxidation compounds, and delay, with respect to the formation of the former, that of some secondary or further oxidation compounds. For this reason, the use of only one or two classical parameters cannot be sufficient to elucidate the behaviour of this compound and perhaps of others able to act as antioxidants.

### 3.5. Importance of Oxylipins Detection and Quantification to Evaluate Biological Activities

The abovementioned oxylipins can be present in oxidized foods, and consequently, they can be absorbed after digestion, but as mentioned before, they can also be formed endogenously by the action of several enzymatic systems. In fact, it has been reported that some oxylipins play important roles in living organisms, and others show varied biological activities, although many remain to be elucidated [91,112].

In the early studies about this subject, the toxic effects provoked by the mixture of lipid oxidation compounds was called oxygen toxicity, and it was attributed to the mixture of unidentified compounds coming from oxidation, including hydroperoxides [113].

Watanabe and Nakamura [114] and Shiotani et al. [115] showed that purified methyl linoleate monohydroperoxide, without specifying the kind of hydroperoxide, has no effect on respiratory or phosphorylating activities of rat heart and liver mitochondria in a certain concentration range. More recently, 13-HPO-9*Z*,11*E*-octadecadienoic acid has been attributed to the ability to relax canine circumflex and splenic arteries [116] and also to promote intestinal inflammation [117]. The ability to activate the peroxisome proliferator-activated receptor α (PPARα) has also been attributed to this same hydroperoxide, leading to a reduction in triglyceride concentrations in liver, plasma and very low-density proteins in rats, but not in human HepG2 hepatoma cells [118]. Nevertheless, although this hydroperoxide was indeed present in the aforementioned assays, the presence of other oxylipins could not be discarded in these experiments, the compound or compounds responsible for the observed biological effect remaining unclear up to a certain point. However, recent studies carried out with pure 9-HPO-10*E*,12*Z*-octadecadienoic acid have shown that this compound plays an effector role in cellular oxidative reactions [119].

Likewise, mHPO-mEPO-mE has shown inhibitory and uncoupling effects on respiratory and phosphorylating activities of rat heart and liver mitochondria [120]. In addition, a great number of bioactive effects have been attributed to some mHO-c-dEs, such as 9-HO-10*E*,12*Z*-dE and 13-HO-9*Z*,11*E*-dE, among which cytotoxic activity and the development and progression of rheumatoid arthritis can be cited [91,121,122]. It is worth noting that, although some of the attributed bioactive effects are produced by both isomers mentioned above, others are caused by only one of them.

In the same way, several biological activities have been attributed to 9-KO- and 13-KO-c-dEs [91], without specifying isomerism, including for the latter compounds the ability to reduce inflammation in human colonic epithelial cells [123]. Furthermore, it is worth noting that it has also been described that 9-KO-10*E*,12*E*-dE induces apoptotic cell death in human ovarian cancer to a greater degree than 9-KO-10*E*,12*Z*-dE [124,125].

Recently, it has been proved that several vicinal and non-vicinal mKO- and mHO-mEPO-mEs fatty acids and their corresponding esterified counterparts are present in normal and psoriatic human skin, the concentration of the modified fatty acids being much higher in psoriatic skin than that of the modified fatty esters [70]. Likewise, potential relationships between the presence of this kind of compound and itchiness, chronic headaches and facial pain have been subject of study [126]. Although these studies constitute outstanding contributions to this issue, it must not be forgotten that, in addition to these oxylipins, many others can also be present in the system, and perhaps the effects are not only due to one group of compounds but to all of them. Finally, regarding mKO-mEPO-mEs, it should be pointed out that 9-KO-12,13-*E*-EPO-10*E*-mE has been considered responsible for the excessive production of aldosterone in visceral obesity and for hypertension in humans [127].

Other important oxidation compounds that can be formed in foods, as has been shown in this study, and also endogenously, are different types of aldehydes. Among them, oxygenated alpha, beta unsaturated aldehydes are considered to be causal agents for different types of degenerative diseases such as Alzheimer, Parkinson, chronic inflammation, adult respiratory distress syndrome, atherogenesis, diabetes, and different types of cancer [80,128,129,130]. Nevertheless, some of them, such as 4-HO-2*E*-nonenal at low concentrations, display several activities referring to cell multiplication and differentiation, stimulation of neutrophil chemotaxis, or activation of membranes, among others [75,76,77,78,79,80].

Likewise, the toxicity of mEPO-mEs that can be formed either in foods or endogenously, in the absence of enzymes or in the presence of cytochrome P450 and epoxygenase, is well known. These compounds, also named leukotoxin and isoleukotoxin, have been considered responsible for acute respiratory distress (ARDS) [131] as well as for circulatory shock and disseminal intravascular coagulation [132]. Furthermore, the oxirane ring opening of these compounds in the presence or absence of epoxide hydrolase leads to the formation of the well-known leukotoxindiol and isoleukotoxindiol. These compounds have been associated with multi-organ failure [100,133], as well as with the development or aggravation of some diseases such as acute respiratory distress syndrome (ARDS) [100,134] and to breast and prostate cancer cell proliferation (in vitro and in vivo) [135].

Lastly, the opening of the epoxide ring in the presence of acids gives rise, as mentioned above, to the formation of additional ester groups that generate branching in modified fatty acyl group chains, which increases the viscosity of the system. When the oxirane ring is opened by alcohol groups, if these are on modified fatty acyl groups, the formed ether bonds could provoke the polymerization of the system.

Lipid oxidation, regardless of whether it is produced enzymatically or not, either in foods or endogenously in humans, is very complex, and many different oxidation derivatives are present in the lipid systems in any oxidation stage, except in the very early oxidation stages. Although the study of biological activity of pure oxidation compounds is very important, the biological activity of mixtures cannot always be deduced from the individual biological activity of their components due either to synergic or antagonist effects. Therefore, it is very important, as some authors [136] already point out, to have techniques and methods that allow the detection and quantification of the largest possible number of oxylipins present in a system in the simplest possible way, in order to establish solid relationships between their composition and concentration and the biological effects they provoke. This study provides both the technique and the methodology to carry out these types of determinations. To do so, one only requires the extraction of the lipid system subject of study, acquiring the ^1^H NMR spectrum and identifying and quantifying the detected oxylipins in the same way as has been reported here. The association of the identity and concentrations of all oxylipins detected in a sample with their overall biological activity observed could contribute to a deeper understanding of this important issue, related to human health.

## 4. Conclusions

^1^H NMR spectroscopy has proven to be a very valuable tool, providing information on oxidative stability, polyunsaturated acyl groups degradation kinetics and kinetics of the formation of a very large number of oxylipins, throughout the oxidation process of walnut oil, alone or enriched with two different concentrations of alpha-tocopherol (αT), submitted to accelerated storage conditions at two different temperatures. Additionally, throughout storage, the degradation kinetic of the added αT has also been addressed. With all this information, it has been possible to elucidate the effects that both the degree of enrichment in αT and the storage temperature provoke in all the aforementioned aspects of the walnut oil oxidation process, which could be extrapolated to any oil rich in polyunsaturated omega-6 groups.

It has been shown that αT in the concentrations tested and at both temperatures acts as a pro-oxidant, reducing the oxidative stability of walnut oil. This is demonstrated by the greater and earlier degradation rate of the polyunsaturated acyl groups of the oil samples enriched with both αT concentrations at both temperatures compared to those of the unenriched sample and by the greater and earlier formation rate of monohydroperoxy conjugated *Z*,*E*-dienes. From the results obtained here, it can be inferred that the enrichment of edible oils with αT under the “*quantum satis*” principle, as is allowed by European legislation [35], can negatively affect the oil oxidative stability if the αT enrichment level is not adequately selected.

In the oxidation undergone during storage by walnut oil without being enriched in αT at low temperature, a very broad induction period occurs, after which the oxidation evolves very quickly, always simultaneously maintaining the formation of monohydroperoxy conjugated *Z*,*E*-dienes and monohydroperoxy conjugated *E*,*E*-dienes, the latter always being of higher concentration than the former. With increasing temperature, the induction period is sharply shortened, but the evolution of the oxidation is similar to at low temperature, although primary oxidation compounds reach lower concentrations.

In the walnut oil enriched with the lowest concentration of αT submitted to low temperature, both primary and secondary or further oxidation compounds are formed earlier than in the non-enriched walnut oil sample subjected to the same storage conditions. Furthermore, unlike in the latter sample, monohydroperoxy conjugated *Z*,*E*-dienes are formed earlier and reach a much higher concentration than their *E*,*E* counterparts. In walnut oil enriched with the highest concentration of αT submitted to low temperature, monohydroperoxy conjugated *Z*,*E*-dienes and secondary or further oxidation compounds with *Z*,*E* isomerism are formed earlier and reach much higher concentrations than in the other two walnut oil samples. In this case, there is also an important delay in the formation of monohydroperoxy conjugated *E*,*E*-dienes compared to that of their *Z*,*E* counterparts that carries over to the formation of most of the oxylipins throughout the storage.

The temperature increase on the walnut oil samples enriched in αT accelerates the oxidation process as expected, although the formation of monohydroperoxide conjugated *Z*,*E*-dienes is earlier in both samples than in the non-enriched sample. In addition, a small delay in the formation of monohydroperoxide conjugated *E*,*E*-dienes with respect to their *Z*,*E* counterparts is observed in the sample less enriched in αT, this delay being more important in the more enriched sample. As consequence, in the latter sample, the formation of most of the secondary or further oxidation compounds occurs later than in the other two samples, and moreover, they reach lower concentrations. It has been shown for the first time that although both αT enrichment and temperature increase, accelerating the oxidation of walnut oil, the former leads to the formation of oxylipins in a very different way to the latter. From the results obtained, it is clear that, due to the complex effect that αT enrichment provokes on the oil oxidation during accelerated storage conditions, the use of classical parameters based on the concentration of secondary or further oxidation compounds can lead to erroneous conclusions on the qualification of the behaviour of the compound tested as an antioxidant or pro-oxidant.

The results obtained have shown that the ingestion of oxidized foods can lead to the absorption of a large number of oxylipins, many of which are the same as those formed endogenously by the action of lipooxygenases, cytochrome P450, epoxygenases or epoxy hydrolases on the same substrates, and as reported, can provoke a large number of negative biological effects of great importance from the point of view of human health.

The method used and described in this study, based on ^1^H NMR spectroscopy, has proven to be very valuable for delving into the oxidation processes of lipids rich in polyunsaturated omega-6 groups occurring under any type of conditions, and consequently to analyze the factors governing the process, which determine the type and the concentrations of the oxylipins present in a lipid system at each stage of an oxidation process. Therefore, this technique can also be considered essential both for a proper evaluation of antioxidant strategies in food systems and for identifying the oxylipins responsible for the negative effects or, where appropriate, for physiological functions in biological systems.

## Figures and Tables

**Table 1:** Degradation rates (D_R_) of the most unsaturated acyl groups (linoleic and linolenic acyl groups) expressed in mmol mol^−1^ TG day^−1^, in the different samples submitted to storage at both temperatures, in each one of their stages. The degradation rates agree with the slopes of the linear equations that relate the concentration of these acyl groups and the storage time. The correlation coefficients of these lines are given in brackets.

Storage Conditions	Acyl Groups	Oil Samples	First Stage		Second Stage	
			Time (Days)	D_R_ (mmol/mol TG Day)	Time (Days)	D_R_ (mmol/mol TG Day)
40 °C	Linoleic	W	0–45	−2.98 (0.95)	45–59	−82.98 (0.99)
		W_02_	0–30	−8.93 (0.92)	30–45	−64.97 (0.98)
		W_2_	0–45	−17.93 (0.97)	45–59	−43.92 (0.95)
	Linolenic	W	0–45	−0.78 (0.91)	45–59	−18.34 (0.99)
		W_02_	0–30	−3.34 (0.92)	30–45	−14.74 (0.97)
		W_2_	0–59	−4.94 (0.99)	---	---
70 °C	Linoleic	W	0–4	−42.91(0.93)	4–8	−264.21 (0.94)
		W_02_	0–4	−57.15 (0.93)	4–8	−247.96 (0.99)
		W_2_	0–8	−66.61 (0.98)	8–12	−187.23 (0.99)
	Linolenic	W	0–4	−3.93 (0.93)	4–7	−77.46 (0.98)
		W_02_	0–4	−12.03 (0.93)	4–8	−58.90 (0.98)
		W_2_	0–12	−21.11 (0.99)	---	---

## Data Availability

The data presented in this study are available in the article and supplementary material.

## Supplementary Materials

The following supporting information can be downloaded at: https://www.mdpi.com/article/10.3390/antiox11040604/s1, Table S1: ^1^H NMR signals, obtained in CDCl_3_, of protons of main walnut oil components shown in Figure 1, their chemical shifts, multiplicities and assignments to protons of different functional groups present in edible oils. The signal letters agree with those given in the Figure 1; Table S2: Chemical shift assignments and multiplicities of ^1^H NMR signals in CDCl_3_ of protons of monohydroperoxy conjugated octadecadienes (mHPO-c-dEs) coming from linoleic groups; Table S3: Chemical shift assignments of ^1^H NMR signals in CDCl_3_ of protons of monohydroperoxy conjugated octadecatrienes (mHPO-c(*Z*,*E*)-tEs) coming from linolenic groups; Table S4: Chemical shift assignments and multiplicities of ^1^H NMR signals in CDCl_3_ of protons of dihydroperoxy non-conjugated *E*,*E*-octadecadienes (dHPO-nc(*E*,*E*)-dEs); Table S5: Chemical shift assignments and multiplicities of ^1^H NMR signals in CDCl_3_ of protons of monohydroperoxy monoepoxy *E*-octadecamonoenes (mHPO-mEPO-*E*-mEs); Table S6: Chemical shift assignments and multiplicities of ^1^H NMR signals in CDCl_3_ of protons of monohydroxy conjugated *Z*,*E*-octadecadienes (mHO-c(*Z*,*E*)-dEs); Table S7: Chemical shift assignments and multiplicities of ^1^H NMR signals in CDCl_3_ of protons of monohydroxy monoepoxy *E*-octadecamonoenes (mHO-mEPO-*E*-mEs); Table S8: Chemical shift assignments and multiplicities of ^1^H NMR signals in CDCl_3_ of protons of monoketo conjugated octadecadienes (mKO-c-dEs); Table S9: Chemical shift assignments and multiplicities of ^1^H NMR signals in CDCl_3_ of protons of monoketo monoepoxy *E*-octadecamonoenes (mKO-mEPO-*E*-mEs); Table S10: Chemical shift assignments and multiplicities of ^1^H NMR signals in CDCl_3_ of protons of different types of aldehydes; Table S11: Chemical shift assignments and multiplicities of ^1^H NMR signals in CDCl_3_ of protons of furan groups; Table S12: Chemical shift assignments and multiplicities of ^1^H NMR signals in CDCl_3_ of protons of epoxy derivatives coming from linoleic acyl groups; Table S13: Chemical shift assignments and multiplicities of ^1^H NMR signals in CDCl_3_ of protons of epoxy derivatives coming from linolenic acyl groups; Table S14: Chemical shift assignments and multiplicities of ^1^H NMR signals in CDCl_3_ of protons of different types of dihydroxy groups (dHO); Table S15: Chemical shift assignments and multiplicities of ^1^H NMR signals in CDCl_3_ of protons of formats; Table S16: Chemical shift assignments and multiplicities of ^1^H NMR signals in CDCl_3_ of protons of alpha-tocopherol.
