# Supplementary material for: Alpha-Tocopherol, a Powerful Molecule, Leads to the Formation of Oxylipins in Polyunsaturated Oils Differently to the Temperature Increase: A Detailed Study by Proton Nuclear Magnetic Resonance of Walnut Oil Oxidation
Source: Antioxidants (Basel). 2022 Mar 22;11(4):604. doi: 10.3390/antiox11040604 (PMC9031923; doi:10.3390/antiox11040604)
Supplement: Supplementary file 1 [file antioxidants-11-00604-s001.zip › antioxidants-1597340-supplementary.pdf]

SUPPLEMENTARY MATERIAL

Alpha-Tocopherol, a Powerful Molecule, Leads to the Formation of Oxylipins in Polyunsaturated Oils Differently to the Temperature Increase. A Detailed Study by Proton Nuclear Magnetic Resonance of Walnut Oil Oxidation

Sofía del Caño-Ochoa, Ainhoa Ruiz-Aracama, María D Guillén *

Food Technology. Faculty of Pharmacy. Lascaray Research Center. University of the Basque Country (UPV/EHU). Paseo de la Universidad nº 7, 01006 Vitoria, Spain, Telf: 34-945-013081. E-mail: mariadolores.guillen@ehu.eus

Table S1. ^{1}H NMR signals, obtained in $CDCl_3$, of protons of main walnut oil components shown in Figures 1, their chemical shifts, multiplicities and assignments to protons of different functional groups present in edible oils. The signal letters agree with those given in the Figure 1.

Signal	Chemical shift (ppm) [a, **]	Multiplicity	Functional group	
			Type of protons *	Compound
A	0.83–0.93	t	-C**H**$_3$	Saturated, oleic and linoleic acyl group
B	0.93–1.03	t	-C**H**$_3$	Linolenic acyl group
C	1.19–1.42	m	-(C**H**$_2$)$_n$-	Acyl groups
D	1.52–1.70	m	-OCO-CH$_2$-C**H**$_2$-	Acyl groups
E	1.94–2.14	m	-C**H**$_2$-CH=CH-	Acyl groups
F	2.23–2.36	dt	-OCO-C**H**$_2$-	Acyl groups
G	2.70–2.84	t	=CH-C**H**$_2$-CH=	Acyl groups
H	4.10–4.32	dd,dd	-C**H**$_2$OCOR	Glyceryl groups
I	5.20–5.29	m	-C**H**OCOR	Glyceryl groups
J	5.29–5.46	m	-C**H**=C**H**-	Acyl groups

Abbreviations: dd: double doublet; t: triplet; dt: double triplet; m: multiplet.

*Area of the signals due to the protons in bold were used for the quantification of each compound.

**The assignment of the ^{1}H NMR signals of the protons was made with the aid of standard compounds.

The assignment of the ^{1}H NMR signals of the protons of main components was made with the data taken from the literature:

[a] Guillén, M.D.; Ruiz, A. (2003). *Eur. J. Lipid Sci. Tech. 105*(11), 688-696.

Table S2. Chemical shift assignments and multiplicities of ^{1}H NMR signals in $CDCl_3$ of protons of monohydroperoxy conjugated octadecadienes (mHPO-c-dEs) coming from linoleic groups.

mHPO-c(*Z*,*E*)-dEs
(9-hydroperoxy-10*E*,12*Z*-octadecadienoate; 13-hydroperoxy-9*Z*,11*E*-octadecadienoate)

Chemical shift (ppm) [a,**]	Multiplicity	Functional group (Type of protons) [*]
**6.58**	dddd	-C**H**=CH-CHOOH-
6.00	ddtd	-CH=CH-CH=CH-CHOOH-
5.56	ddm	-CH=CH-CHOOH-
5.51	dtm	-CH=CH-CH=CH-CHOOH-
4.37	dtd	-CHOOH-
2.30	t	-OCO-CH$_2$-
2.19	dtd	-CH$_2$-CH=CH-
1.55–1.71	m	-CHOOH-CH$_2$-<br>-OCO-CH$_2$-CH$_2$-
1.24–1.43	m	-CH$_2$-
0.89	t	-CH$_3$

mHPO-c(*E*,*E*)-dEs
(9-hydroperoxy-10*E*,12*E*-octadecadienoate; 13-hydroperoxy-9*E*,11*E*-octadecadienoate)

Chemical shift (ppm) [a,**]	Multiplicity	Functional group (Type of protons) [*]
**6.27**	ddm	-C**H**=CH-CHOOH-
6.06	ddtd	-CH=CH-CH=CH-CHOOH-
5.76	dtm	-CH=CH-CH=CH-CHOOH-
5.47	ddm	-CH=CH-CHOOH-
4.32	dtd	-CHOOH-
2.30	t	-OCO-CH$_2$ -
2.09	dtd	-CH$_2$-CH=CH-
1.42–1.66	m	-CHOOH-CH$_2$-<br>-OCO-CH$_2$-CH$_2$ -
1.25–1.43	m	-CH$_2$-
0.89	t	-CH$_3$

Abbreviations: dddd: double of double of double doublet; ddtd: double of double of triple doublet; dtd: double of triple doublet; t: triplet; m: multiplet; ddm: double of double multiplet; dtm: double of triple multiplet.

[*]Area of the signals due to the protons in bold, together with the area of the *sn*-1 and *sn*-3 signals of TG shown, in Table S1 and in Figure 1, were used for the quantification of each compound, using the equation [eq. 1] showed in the Materials and Methods.

[**]The assignment of the ^{1}H NMR signals of the protons was made with the aid of standard compounds.

The assignment of the ^{1}H NMR signals of the protons of mHPO-c-dEs derived from linoleic acyl groups was made with the data taken from the literature:

[a] Goicoechea, E.; Guillén, M.D. (2010). *J. Agric. Food Chem. 58*, 6234-6245.

[a] Pajunen, T.I.; Koskela, H.; Hase, T.; Hopia, A. (2008). *Chem. Phys. Lipids. 154*, 105-114.

Table S3. Chemical shift assignments of ^{1}H NMR signals in $CDCl_3$ of protons of monohydroperoxy conjugated octadecatrienes (mHPO-c(*Z*,*E*)-tEs) coming from linolenic groups.

**mHPO-c(*Z*,*E*)-tEs (16-hydroperoxy-9*Z*,12*Z*,14*E*-octadecatrienoate; 9-hydroperoxy-10*E*,12*Z*,15*Z*-octadecatrienoate)**	
**Chemical shift (ppm)** [a]	**Functional group (Type of protons)** *
**6.61**	-C**H**=CH-CHOOH-
6.03	-CH=CH-CH=CH-CHOOH-
5.61	-CH=CH-CHOOH-
5.47	-CH=CH-CH=CH-CHOOH-
5.42	-CH=CH-CH_2-CH=CH-CH=CH-CHOOH-
5.36	-CH=CH-CH_2-CH=CH-CH=CH-CHOOH-
4.34	-CHOOH-
2.95	-CH=CH-CH_2-CH=CH-
2.30	-OCO-CH_2-
2.05	-CH_2-CH_2-CH=CH-
1.55–1.72	-CH_2-CHOOH-
1.2–1.8	-CH_2-
0.95	-CH_3

* Area of the signals due to the protons in bold, together with the area of the *sn*-1 and *sn*-3 signals of TG shown, in Table S1 and in Figure 1, were used for the quantification of each compound, using the equation [eq. 1] showed in the Materials and Methods.

The assignment of the ^{1}H NMR signals of the protons of mHPO-c(*Z*,*Z*,*E*)-tEs was made with the data taken from the literature:

[a]Ahmed, R.; Panayiotis, C.V.; Siskos, M.G; Siddiqui, H.; Choudhary, M.I.; Gerothanassis, P. (2020). *Molecules, 25*, 4902.

[a] Neff, W.E.; Frankel, E.N.; Fujimoto, K. (1988). *J. Am. Oil Chem. Soc. 65*(4), 616-623.

Table S4. Chemical shift assignments and multiplicities of ^{1}H NMR signals in $CDCl_3$ of protons of dihydroperoxy non conjugated *E,E*-octadecadienes (dHPO-nc(*E,E*)-dEs).

dHPO-nc(*E,E*)-dEs (9,12-dihydroperoxy-10*E*,13*E*-octadecadienoate; 10,13-dihydroperoxy-8*E*,11*E*-octadecadienoate)		
**Chemical shift (ppm)** [a]	**Multiplicity**	**Functional group (Type of protons)** *
5.56–5.87	m	-CHOOH-CH=CH-CHOOH-<br>-CHOOH-CH=CH-CH_2-
5.36–5.50	m	-CHOOH-CH=CH-CH_2-
**4.81**	dd	-C**H**OOH-
4.30–4.39	m	-CHOOH-
2.33	t	-OCO-CH_2-
2.02–2.11	m	-CH=CH-CH_2-
1.54–1.69	m	-OCO-CH_2-CH_2-
1.16–1.43	m	-CH_2-
0.81–0.91	t	-CH_3

Abbreviations: dd: double doublet; t: triplet; m: multiplet.

*Area of the signals due to the protons in bold, together with the area of the *sn*-1 and *sn*-3 signals of TG shown, in Table S1 and in Figure 1, were used for the quantification of each compound, using the equation [eq. 1] showed in the Materials and Methods.

The assignment of the ^{1}H NMR signals of the protons of dHPO-nc(*E,E*)-dEs was made with the data taken from the literature:

[a] Zhang, W. (2008). Synthesis and Fragmentation Reactions of Linoleic Acid-Derived Hydroperoxides (Doctoral dissertation, Case Western Reserve University).

Table S5. Chemical shift assignments and multiplicities of ^{1}H NMR signals in $CDCl_3$ of protons of monohydroperoxy monoepoxy *E*-octadecamonoenes (mHPO-mEPO-*E*-mEs).

Chemical shift (ppm) [a]	Multiplicity	Functional group (Type of protons) *
		**mHPO-*E*-mEPO-*E*-mEs (9-hydroperoxy-12,13-*E*-epoxy-10*E*-octadecenoate)**
**5.85**	dd	-CHOCH-CH=C**H**-CHOOH-
5.47	dd	-CHOCH-CH=CH-CHOOH-
4.33	m	-CHOOH-
3.11	dd	-CHOCH-CH=CH-
2.84	m	-CHOCH-CH=CH-
2.30	t	-OCO-CH_2-
1.51	m	-OCO-CH_2-CH_2-
1.31	m	-CH_2-
0.89	t	-CH_3

Chemical shift (ppm) [b]	Multiplicity	Functional group (Type of protons) *
		**mHPO-*Z*-mEPO-*E*-mEs (13-hydroperoxy-9,10-*Z*-epoxy-11*E*-octadecenoate)**
**5.84**	dd	-CHOCH-CH=C**H**-CHOOH-
5.5–5.7	m	-CHOCH-CH=CH-CHOOH-
4.2–4.4	m	-CHOOH-
3.42	dd	-CHOCH-CH=CH-
3.0–3.2	m	-CHOCH-CH=CH-
2.32	t	-OCO-CH_2-
1.1–1.8	m	-CH_2-
0.85	t	-CH_3

Abbreviations: dd: double doublet; t: triplet; m: multiplet.

* Area of the signals due to the protons in bold, together with the area of the *sn*-1 and *sn*-3 signals of TG shown, in Table S1 and in Figure 1, were used for the quantification of each compound, using the equation [eq. 1] showed in the Materials and Methods.

The assignment of the ^{1}H NMR signals of the protons of mHPO-mEPO-*E*-mEs was made with the data taken from the literature:

[a] Gardner, H.W.; Weisleder, D.; Kleiman, R. (1978). *Lipids, 13*(4), 246-252.

[b] Gu, X.; Salomon, R. (2012). *Free Radical Biol. Med. 52*, 601-606.

Table S6. Chemical shift assignments and multiplicities of ^{1}H NMR signals in $CDCl_3$ of protons of monohydroxy conjugated *Z*,*E*-octadecadienes (mHO-c(*Z*,*E*)-dEs).

**mHO-c(*Z*,*E*)-dEs (13-hydroxy-9*Z*,11*E*-octadecadienoate; 9-hydroxy-10*E*,12*Z*-octadecadienoate)**		
**Chemical shift (ppm)** [a,**]	**Multiplicity**	**Functional group (Type of protons)** *
**6.46**	ddd	-C**<u>H</u>**=CH-CHOOH-
5.94	dd	-CH=C<u>H</u>-CH=CH-CHOOH-
5.64	dd	-CH=C<u>H</u>-CHOOH-
5.42	ddt	-C<u>H</u>=CH-CH=CH-CHOOH-
4.13	m	-C<u>H</u>OH-
2.27	t	-OCO-C<u>H</u>$_2$-
2.15	m	-CH=CH-C<u>H</u>$_2$-
1.2–1.7	m	-C<u>H</u>$_2$-
0.86	t	-C<u>H</u>$_3$

Abbreviations: dd: double doublet; ddd: double of double doublet; t: triplet; ddt: double of double triplet; m: multiplet.

[*] Area of the signals due to the protons in bold, together with the area of the *sn*-1 and *sn*-3 signals of TG shown, in Table S1 and in Figure 1, were used for the quantification of each compound, using the equation [eq. 1] showed in the Materials and Methods.

[**] The assignment of the ^{1}H NMR signals of the protons was made with the aid of standard compounds.

The assignment of the ^{1}H NMR signals of the protons of mHO-c(*Z*,*E*)-dEs was made with the data taken from the literature:

[a] Kuklev, D. V.; Christie, W. W.; Durand, T.; Rossi, J. C.; Vidal, J. P.; Kasyanov, S. P.;... Bezuglov, V. V. (1997). *Chem. Phys. Lipids, 85*(2), 125-134.

Table S7. Chemical shift assignments and multiplicities of ^{1}H NMR signals in $CDCl_3$ of protons of monohydroxy monoepoxy *E*-octadecamonoenes (mHO-mEPO-*E*-mEs).

**mHO-*E*-mEPO-*E*-mEs (9-hydroxy-12,13-*E*-epoxi-10*E*-octadecenoate; 13-hydroxy-9,10-*E*-epoxy-11*E*-octadecenoate[**])**		
**Chemical shift (ppm)[a]**	**Multiplicity**	**Functional group (Type of protons)[*]**
**5.93**	dd	-CH=C**H**-CHOH-
5.41	ddd	-CH=CH-CHOH-
4.14	m	-CHOH-
3.09	dd	-CHOCH-CH=CH-
2.81	dt	-CHOCH-CH=CH-
2.30	t	-OCO-CH_2-
1.49	m	-OCO-CH_2-CH_2-
1.31	m	-CH_2-
0.89	t	-CH_3
**mHO-*Z*-mEPO-*E*-mEs (13-hydroxy-9,10-Z-epoxy-11*E*-octadecenoate)**		
**Chemical shift (ppm)[b]**	**Multiplicity**	**Functional group (Type of protons)[*]**
**5.95**	dd	-CH=C**H**-CHOH-
5.54	ddd	-CH=CH-CHOH-
3.41	dd	-CHOCH-CH=CH-
3.07	dt	-CHOCH-CH=CH-
2.30	t	-OCO-CH_2-
2.10	br	-CHOH-
1.2–1.6	m	-CH_2-
0.89	t	-CH_3

Abbreviations: dd: double doublet; ddd: double of double doublet; t: triplet; dt: double triplet; m: multiplet; br: broad signal.

[*] Area of the signals due to the protons in bold, together with the area of the *sn*-1 and *sn*-3 signals of TG shown, in Table S1 and in Figure 1, were used for the quantification of each compound, using the equation [eq. 1] showed in the Materials and Methods.

[**] The assignment of the ^{1}H NMR signals of the protons was made with the aid of standard compounds.

The assignment of the ^{1}H NMR signals of the protons of mHO-mEPO-*E*-mEs was made with the data taken from the literature:

[a] Gardner, H.W.; Weisleder, D.; Kleiman, R. (1978). *Lipids, 13*(4), 246-252.

[a] Schieberle, P.; Trebert, Y.; Firl, J.; Grosch, W. (1988). *Chem. Phys. Lipids, 48*(3-4), 281-288.

[a] Ramsden, C.E.; Domenichiello, A.F.; Yuan, Z.X.; Sapio, M.R.; Keyes, G.S.; Mishra, S. K.; ... Davis, J.M. (2017). *Sci. Sign. 10*(493), eaal5241.

[b] Hidalgo, F.J.; Zamora, R.; Vioque, E. (1992). *Chem. Phys. Lipids, 60*(3), 225-233.

Table S8. Chemical shift assignments and multiplicities of ^{1}H NMR signals in $CDCl_3$ of protons of monoketo conjugated octadecadienes (mKO-c-dEs).

**mKO-c(*Z*,*E*)-dEs (13-keto-9*Z*,11*E*-octadecadienoate; 9-keto-10*E*,12*Z*-octadecadienoate)**		
**Chemical shift (ppm) [a, **]**	**Multiplicity**	**Functional group (Type of protons) [*]**
**7.49**	ddd	-CO-CH=C**H**-
6.16	d	-CO-CH=CH-
6.12	dd	-CO-CH=CH-CH=CH-
5.91	dt	-CO-CH=CH-CH=CH-
2.55	t	-CH_2-CO-
2.34	t	-OCO-CH_2-
2.30	m	-CH=CH-CH_2-
1.32–1.63	m	-CH_2-
0.88	t	-CH_3
**mKO-c(*E*,*E*)-dEs (13-keto-9*E*,11*E*-octadecadienoate; 9-keto-10*E*,12*E*-octadecadienoate)**		
**Chemical shift (ppm) [a, **]**	**Multiplicity**	**Functional group (Type of protons) [*]**
**7.13**	dm	-CO-CH=C**H**-
6.14–6.19	m	-CO-CH=CH-CH=CH-
6.07	d	-CO-CH=CH-
2.53	t	-CH_2-CO-
2.35	t	-OCO-CH_2-
2.17	m	-CH=CH-CH_2-
1.32–1.63	m	-CH_2-
0.88	t	-CH_3

Abbreviations: d: doublet; dd:double doublet; ddd; double of double doublet; t: triplet; dt: double triplet; m: multiplet; dm: double multiplet.

[*] Area of the signals due to the protons in bold, together with the area of the *sn*-1 and *sn*-3 signals of TG shown, in Table S1 and in Figure 1, were used for the quantification of each compound, using the equation [eq. 1] showed in the Materials and Methods.

[**] The assignment of the ^{1}H NMR signals of the protons was made with the aid of standard compounds.

The assignment of the ^{1}H NMR signals of the protons of mKO-c-dEs was made with the data taken from the literature:

[a] Dufour, C.; Loonis, M. (2005). *Chem. Phys. Lipids. 138*(1), 60-68.

Table S9. Chemical shift assignments and multiplicities of ^{1}H NMR signals in $CDCl_3$ of protons of monoketo monoepoxy *E*-octadecamonoenes (mKO-mEPO-*E*-mEs).

***non vicinal*-mKO-*Z*-mEPO-*E*-mEs (13-keto-9,10-*Z*-epoxy-11*E*-octadecenoate; 9-keto-12,13-*Z*-epoxy-10*E*-octadecenoate)**		
**Chemical shift (ppm)** [a]	**Multiplicity**	**Functional group (Type of protons)** [*]
**6.66**	dd	-CO-CH=C**H**-
6.40	d	-CO-CH=CH-
3.52	dd	-CH=CH-CHOCH-
3.20	dd	-CH=CH-CHOCH-
2.55	t	-CH_2-CO-
2.30	t	-OCO-CH_2-
1.2–1.7	m	-CH_2-
0.89	t	-CH_3
***non vicinal*-mKO-*E*-mEPO-*E*-mEs (13-keto-9,10-*E*-epoxy-11*E*-octadecenoate; 9-keto-12,13-*E*-epoxy-10*E*-octadecenoate)**		
**Chemical shift (ppm)** [a,**]	**Multiplicity**	**Functional group (Type of protons)** [*]
6.52	dd	-CO-CH=CH-
6.38	d	-CO-CH=CH-
**3.21**	dd	-CH=CH-C**H**OCH-
2.89	td	-CH=CH-CHOCH-
2.53	t	-CH_2-CO-
2.30	t	-OCO-CH_2-
1.2–1.6	m	-CH_2-
0.86	t	-CH_3
***vicinal*-mKO-*E*-mEPO-*E*-mEs (11-keto-12,13-*E*-epoxy-9*E*-octadecenoate; 11-keto-9,10-*E*-epoxy-12*E*-octadecenoate)**		
**Chemical shift (ppm)** [b]	**Multiplicity**	**Functional group (Type of protons)** [*]
**7.02**	dt	-CO-CH=C**H**-
6.16–6.23	dt	-CO-CH=CH-
3.28–3.34	d	-CHOCH-CO-
2.98–3.04	ddd	-CHOCH-CO-
2.29–2.34	t	-OCO-CH_2-
2.16–2.22	dtd	-CH=CH-CH_2-
1.25–1.70	m	-CH_2-
0.82–0.89	t	-CH_3

Abbreviations: d: doublet; dd:double doublet; td: triple doublet; ddd:double of double doublet; dtd: double of triple doublet; t: triplet; dt: double triplet; m: multiplet.

[*] Area of the signals due to the protons in bold, together with the area of the *sn*-1 and *sn*-3 signals of TG shown, in Table S1 and in Figure 1, were used for the quantification of each compound, using the equation [eq. 1] showed in the Materials and Methods.

[**] The assignment of the ^{1}H NMR signals of the protons was made with the aid of standard compounds.

The assignment of the ^{1}H NMR signals of the protons of mKO-mEPO-*E*-mEs was made with the data taken from the literature:

[a] Hidalgo, F.J.; Zamora, R.; Vioque, E. (1992). *Chem. Phys. Lipids. 60*(3), 225-233.

[a,b] Lin, D.; Zhang, J.; Sayre, L.M. (2007). *J. Org. Chem. 72*(25), 9471-9480.

[a] Ramsden, C.E.; Domenichiello, A.F.; Yuan, Z.X.; Sapio, M.R.; Keyes, G.S.; Mishra, S.K.; Gross, J.R.; Majchrzak-Hong, S.; Zamora, D.; Horowitz, M. S.; et al. (2017). *Sci. Sign., 10*(493), eaal5241.

[a] Gardner, H.W.; Kleiman, R.; Weisleder, D. (1974). *Lipids, 9* (9), 696-706.

Table S10. Chemical shift assignments and multiplicities of ^{1}H NMR signals in $CDCl_3$ of protons of different types of aldehydes.

***n*-alkanals**		
**Chemical shift (ppm) [a,**]**	**Multiplicity**	**Functional group (Type of protons) [*]**
**9.75**	t	-C$\underline{\mathbf{H}}$O
2.40	dt	CHO-C$\underline{H}_2$-
1.61	m	CHO-CH_2-C$\underline{H}_2$-
1.27–1.32	br	-C$\underline{H}_2$-
0.88	t	-C$\underline{H}_3$
**2*E*-alkenals**		
**Chemical shift (ppm) [a,**]**	**Multiplicity**	**Functional group (Type of protons) [*]**
**9.49**	d	-C$\underline{\mathbf{H}}$O
6.85	tt	CHO-CH=C$\underline{H}$-
6.11	dd	CHO-C$\underline{H}$=CH-
2.32	q	-CH=CH-C$\underline{H}_2$-
1.69–1.19	br	-C$\underline{H}_2$-
0.89	t	-C$\underline{H}_3$
**2*E*,4*E*-alkadienals**		
**Chemical shift (ppm) [a,**]**	**Multiplicity**	**Functional group (Type of protons) [*]**
**9.53**	d	-C$\underline{\mathbf{H}}$O
7.09	m	CHO-CH=C$\underline{H}$-
6.30	m	-CH=C$\underline{H}$-
6.08	dd	CHO-C$\underline{H}$=CH-
2.22	m	-CH=CH-C$\underline{H}_2$-
1.30–1.47	m	-C$\underline{H}_2$-
0.90	t	-C$\underline{H}_3$
**4,5-epoxy-2*E*-alkenals**		
**Chemical shift (ppm) [a,**]**	**Multiplicity**	**Functional group (Type of protons) [*]**
**9.54**	d	-C$\underline{\mathbf{H}}$O
6.56	dd	CHO-CH=C$\underline{H}$-
6.40	dd	CHO-C$\underline{H}$=CH-
3.33	dd	-C$\underline{H}$OCH-
2.96	td	-CHOC$\underline{H}$-
1.33–1.65	m	-C$\underline{H}_2$-
0.91	t	-C$\underline{H}_3$
**4-hydroperoxy-2*E*-alkenals**		
**Chemical shift (ppm) [a,**]**	**Multiplicity**	**Functional group (Type of protons) [*]**
**9.58**	d	-C$\underline{\mathbf{H}}$O
8.20	d	-OO$\underline{H}$
6.80	dd	CHO-CH=C$\underline{H}$-
6.33	m	CHO-C$\underline{H}$=CH-
4.66	dd	-C$\underline{H}$OOH-
1.21–1.73	m	-C$\underline{H}_2$-
0.89	t	-C$\underline{H}_3$
**4-hydroxy-2*E*-alkenals**		
**Chemical shift (ppm) [a,**]**	**Multiplicity**	**Functional group (Type of protons) [*]**
**9.57**	d	-C$\underline{\mathbf{H}}$O
6.84	dd	CHO-CH=C$\underline{H}$-
6.33	dddd	CHO-C$\underline{H}$=CH-
4.43	m	-C$\underline{H}$OH-

1.30–1.70	m	-C$\underline{H}_2$-
0.90	t	-C$\underline{H}_3$
	**2*Z*-alkenals**	
**Chemical shift (ppm)** [b]	**Multiplicity**	**Functional group (Type of protons)** *
**10.06**	d	-C$\underline{\mathbf{H}}$O
	**2*E*,4*Z*-alkadienals**	
**Chemical shift (ppm)** **	**Multiplicity**	**Functional group (Type of protons)** *
**9.60**	d	-C$\underline{\mathbf{H}}$O

Abbreviations: d: doublet; dd:double doublet; td:triple doublet; t: triplet; tt:triple triplet; q: quadruplet; m: multiplet; br: broad signal.

* Area of the signals due to the protons in bold, together with the area of the *sn*-1 and *sn*-3 signals of TG shown, in Table S1 and in Figure 1, were used for the quantification of each compound, using the equation [eq. 1] showed in the Materials and Methods.

** The assignment of the ^{1}H NMR signals of the protons was made with the aid of standard compounds.

The assignment of the ^{1}H NMR signals of the protons of aldehydes was made with the data taken from the literature:

[a] Guillén, M.D.; Ruiz, A. (2004). *Eur. J. Lipid Sci. Tech. 106*(10), 680-687.

[a] Guillén, M.D.; Ruiz, A. (2005a). *Eur. J. Lipid Sci. Tech. 107*(1), 36-47.

[a] Guillén, M.D. ; Ruiz, A. (2005b). *J. Sci. Food Agric. 85*(14), 2413-2420.

[a] Goicoechea, E.; Guillen, M.D. (2010). *J. Agric. Food Chem. 58*(10), 6234-6245.

[b] Moumtaz, S.; Percival, B.C.; Parmar, D.; Grootveld, K.L.; Jansson, P.; Grootveld, M. (2019). *Sci. Rep. 9*, 1-21.

Table S11. Chemical shift assignments and multiplicities of ^{1}H NMR signals in $CDCl_3$ of protons of furan groups.

Chemical shift (ppm) **	Multiplicity	Functional group (Type of protons) *
**Alkyl-furans**		
7.27	dd	-CH=CH- (ar. C-4)
6.24	dd	-CH=CH- (ar. C-3)
5.94	m	-CH- (ar. C-5)
**Alkyl furanones (5-pentyl-(5H)-furan-2-one)**		
**Chemical shift (ppm) [a]**	**Multiplicity**	**Functional group (Type of protons) ***
**7.46**	dd	-CH=C**H**- (ar. C-4)
6.10	dd	-CH=CH- (ar- C-3)
5.04	m	-CH- (ar. C-5)
1.26–2.80	m	-CH_2-
0.90	t	-CH_3

Abbreviations: dd: double doublet; t: triplet; m: multiplet.

* Area of the signals due to the protons in bold, together with the area of the *sn*-1 and *sn*-3 signals of TG shown, in Table S1 and in Figure 1, were used for the quantification of each compound, using the equation [eq. 1] showed in the Materials and Methods.

** The assignment of the ^{1}H NMR signals of the protons was made with the aid of standard compounds.

The assignment of the ^{1}H NMR signals of the protons of 5-pentyl-(5H)-furan-2-one was made with the data taken from the literature:

[a] Bonete, P.; Najera, C. (1994). *J. Org. Chem. 59*(11), 3202-3209.

[a] Braukmüller, S.; Brückner, R. (2006). *Eur. J. Org. Chem. 2006*(9), 2110-2118.

Table S12. Chemical shift assignments and multiplicities of ^{1}H NMR signals in $CDCl_3$ of protons of epoxy derivatives coming from linoleic acyl groups.

	***Z* epoxides coming from linoleic acyl groups (Mono-epoxides)**	
**Chemical shift (ppm)** [a,b,**]	**Multiplicity**	**Functional group (Type of protons)** [*]
5.50–5.60	m	-CHOCH-CH$_2$-CH=CH-
5.35–5.50	m	-CHOCH-CH$_2$-CH=CH-
**2.95**	m	-C**H**OC**H**-CH$_2$-CH=CH-
2.20–2.40	m	-CHOCH-CH$_2$-CH=CH-
1.45–1.60	m	-CH$_2$-CH$_2$-CHOCH-
0.87	t	-CH$_3$
	***Z* epoxides coming from linoleic acyl groups (Di-epoxides)**	
**Chemical shift (ppm)** [a, b, **]	**Multiplicity**	**Functional group (Type of protons)** [*]
3.09–3.14	m	-CHOCH-CH$_2$-CHOCH-
3.00	m	-CHOCH-CH$_2$-CHOCH-
1.70–1.85	m	-CHOCH-CH$_2$-CHOCH-
1.45–1.60	m	-CH$_2$-CH$_2$-CHOCH-
0.87	t	-CH$_3$
	***E* epoxides coming from linoleic acyl groups (Mono-epoxides)**	
**Chemical shift (ppm)** [a, **]	**Multiplicity**	**Functional group (Type of protons)** [*]
5.47–5.56	m	-CHOCH-CH$_2$-CH=CH-
5.33–5.42	m	-CHOCH-CH$_2$-CH=CH-
**2.66–2.71**	m	-C**H**O**H**C-
2.35–2.44	m	-CHOCH-CH$_2$-CH=CH-
1.26–1.66	m	-CH$_2$-
0.87	t	-CH$_3$

Abbreviations: t: triplet; m: multiplet.

[*] Area of the signals due to the protons in bold, together with the area of the *sn*-1 and *sn*-3 signals of TG shown, in Table S1 and in Figure 1, were used for the quantification of each compound, using the equation [eq. 1] showed in the Materials and Methods.

[**] The assignment of the ^{1}H NMR signals of the protons was made with the aid of standard compounds.

The assignment of the ^{1}H NMR signals of the protons of epoxides derived from linoleic acyl groups was made with the data taken from the literature:

[a] Nilewski, C.; Chapelain, C.L.; Wolfrum, S.; Carreira, E.M. (2015). *Org. Lett. 17*(22), 5602-5605.

[b] Xia, W.; Budge, S.M.; Lumsden, M.D. (2016). *J. Am. Oil. Chem. Soc. 93*, 467-478.

Table S13. Chemical shift assignments and multiplicities of ^{1}H NMR signals in $CDCl_3$ of protons of epoxy derivatives coming from linolenic acyl groups.

Z epoxides coming from linolenic acyl groups (Mono-epoxides)		
**Chemical shift (ppm)** [a]	**Multiplicity**	**Functional group (Type of protons)** [*]
5.50–5.60	m	-CHOCH-CH$_2$-CH=CH-
5.35–5.50	m	-CHOCH-CH$_2$-CH=CH-
**2.95**	m	-C**H**OC**H**-CH$_2$-CH=CH-
2.25–2.40	m	-CHOCH-CH$_2$-CH=CH-
1.45–1.60	m	-CH$_2$-CH$_2$-CHOCH-
1.08	m	CH$_3$-CH$_2$-CHOCH-

Z epoxides coming from linolenic acyl groups (Di-epoxides)		
**Chemical shift (ppm)** [a]	**Multiplicity**	**Functional group (Type of protons)** [*]
5.63	m	-CHOCH-CH$_2$-CH=CH-CH$_2$-CHOCH-
5.50–5.60	m	-CHOCH-CH$_2$-CH=CH-
5.35–5.50	m	-CHOCH-CH$_2$-CH=CH-
2.95	m	-CHOCH-CH$_2$-CH=CH-
2.25–2.40	m	-CHOCH-CH$_2$-CH=CH-
1.70–1.85	m	-CHOCH-CH$_2$-CHOCH-
1.45–1.60	m	-CH$_2$-CH$_2$-CHOCH-
1.08	m	CH$_3$-CH$_2$-CHOCH-

Z epoxides coming from linolenic acyl groups (Tri-epoxides)		
**Chemical shift (ppm)** [a]	**Multiplicity**	**Functional group (Type of protons)** [*]
3.16–3.21	m	-CHOCH-CH$_2$-CHOCH-CH$_2$-CHOCH-
3.00	m	-CHOCH-CH$_2$-CHOCH-CH$_2$-CHOCH-
1.70–1.85	m	-CHOCH-CH$_2$-CHOCH-
1.45–1.60	m	-CH$_2$-CH$_2$-CHOCH-
1.08	m	CH$_3$-CH$_2$-CHOCH-

Abbreviations: m: multiplet.

[*] Area of the signals due to the protons in bold, together with the area of the *sn*-1 and *sn*-3 signals of TG shown, in Table S1 and in Figure 1, were used for the quantification of each compound, using the equation [eq. 1] showed in the Materials and Methods.

[**] The assignment of the ^{1}H NMR signals of the protons was made with the aid of standard compounds. The assignment of the ^{1}H NMR signals of the protons of epoxides derived from linolenic acyl groups was made with the data taken from the literature:

[a] Xia, W.; Budge, S.M.; Lumsden, M.D. (2016). *J. Am. Oil. Chem. Soc. 93*, 467-478.

Table S14. Chemical shift assignments and multiplicities of ^{1}H NMR signals in $CDCl_3$ of protons of different types of dihydroxy groups (dHO).

dHO (9,10-dihydroxy-12Z-octadecanoate)		
**Chemical shift (ppm)** [a,**]	**Multiplicity**	**Functional group (Type of protons)** [*]
5.52	m	-C<u>H</u>=CH-CH_2-CHOH-CHOH-
5.42	m	-CH=C<u>H</u>-CH_2-CHOH-CHOH-
**3.42**	br	-C<u>**H**</u>OH-C<u>**H**</u>OH-
2.62	br	-CHO<u>H</u>-CHOH-
2.27	t	-OCO-C<u>H</u>$_2$-
2.25	q	-CH=CH-C<u>H</u>$_2$-CHOH-CHOH-
2.02	q	-C<u>H</u>$_2$-CH=CH-CH_2-CHOH-CHOH-
1.28–1.60	br	-C<u>H</u>$_2$-
0.87	t	-C<u>H</u>$_3$

Abbreviations: t: triplet; q:quadruplet; m: multiplet; br: broad signal.

[*] Area of the signals due to the protons in bold, together with the area of the *sn*-1 and *sn*-3 signals of TG shown, in Table S1 and in Figure 1, were used for the quantification of each compound, using the equation [eq. 1] showed in the Materials and Methods.

[**] The assignment of the ^{1}H NMR signals of the protons was made with the aid of standard compounds.

The assignment of the ^{1}H NMR signals of the protons of dHO was made with the data taken from the literature:

[a] Nilewski, C.; Chapelain, C.L.; Wolfrum, S.; Carreira, E.M. (2015). *Org. Lett. 17*(22), 5602-5605.

[a] Yang, J.; Morton, M.D.; Hill, D.W.; Grant, D.F. (2006). *Chem. Phys. Lipids. 140* (1-2), 75-87.

Table S15. Chemical shift assignments and multiplicities of ^{1}H NMR signals in $CDCl_3$ of protons of formates.

Formates or polyformates		
**Chemical shift (ppm)** [a]	**Multiplicity**	**Functional group (Type of protons)** [*]
**8.05–8.20**	m	$\text{-CH}_2\text{-CH-OC}\underline{\textbf{H}}\text{=O-}$
2.3	t	$\text{-OCO-C}\underline{\text{H}}_2\text{-}$
2.05	s	$\text{-C}\underline{\text{H}}_2\text{-CH-OCH=O-}$
1.3–1.5	m	$\text{-C}\underline{\text{H}}_2\text{-}$
0.9	t	$\text{-C}\underline{\text{H}}_3$

Abbreviations: s: singlet; t: triplet; m: multiplet.

[*] Area of the signals due to the protons in bold, together with the area of the *sn*-1 and *sn*-3 signals of TG shown, in Table S1 and in Figure 1, were used for the quantification of each compound, using the equation [eq. 1] showed in the Materials and Methods.

The assignment of the ^{1}H NMR signals of the protons of formic acid and formates was made with the data taken from the literature:

[a] Harry-O'kuru, R.E.; Biresaw, G.; Tisserat, B.; Evangelista, R. (2016). *J. Lipids, ID 3128604*, 12.

Table S16. Chemical shift assignments and multiplicities of ^{1}H NMR signals in $CDCl_3$ of protons of alpha-tocopherol.

alpha-tocopherol

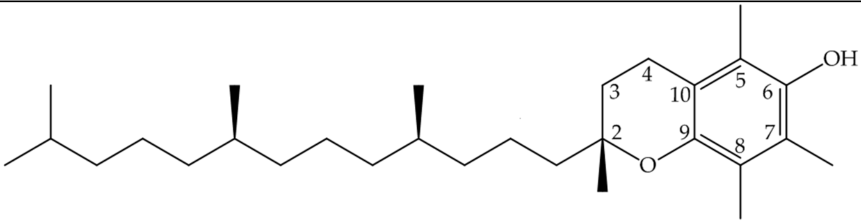

Chemical shift (ppm) [a, **]	Multiplicity	Functional group	Type of protons *
4.18	s	Hydroxy group bonded to C6	-O$\underline{H}$
**2.60**	t	Methylene group in position 4	-C$\mathbf{\underline{H}}_2$-
2.15	s	Methyl group bonded to C7	-C$\underline{H}_3$
2.11	s	Methyl group bonded to C5 and C8	-C$\underline{H}_3$
1.8	m	Methylene group in position 3	-C$\underline{H}_2$-
1.22	s	Methyl group bonded to C2	-C$\underline{H}_3$
1.1–1.5	m	Chain methylene groups<br>Chain methine groups	-C$\underline{H}_2$-<br>-C$\underline{H}$-
0.83–0.88	m	Chain methyl groups	-C$\underline{H}_3$

Abbreviations: s: singlet; t: triplet; m: multiplet.

* Area of the signals due to the protons in bold, together with the area of the *sn*-1 and *sn*-3 signals of TG shown, in Table S1 and in Figure 1, were used for the quantification of each compound, using the equation [eq. 1] showed in the Materials and Methods.

** The assignment of the ^{1}H NMR signals of the protons was made with the aid of standard compounds.

The assignment of the ^{1}H NMR signals of the protons of alpha-tocopherol was made with the data taken from the literature:

[a] Baker, J.K.; Myers, C.W. (1991). *Pharm. Res. 8*(6), 763-770.
